# Correlation Is Not Prediction: Reassessing Predictive MRI Evidence in Guidelines for Persons With Relapsing-Remitting Multiple Sclerosis

**DOI:** 10.1177/11795735261453967

**Published:** 2026-06-25

**Authors:** Dulat Minas, Stefan Buchka, Joachim Havla, Ulrich Mansmann

**Affiliations:** 1 Department of Medical Information Processing, Biometry, and Epidemiology, Medical Faculty, 54187 Ludwig-Maximilian University Munich, Munich, Germany; 2 Pettenkofer School of Public Health, Medical Faculty, 54187 Ludwig-Maximilian University Munich, Munich, Germany; 3 Institute of Clinical Neuroimmunology, LMU Hospital, 54187 Ludwig-Maximilian University Munich, Munich, Germany

**Keywords:** relapsing-remitting multiple sclerosis, prediction, disease-modifying therapy, personalized MS-treatment, treatment monitoring

## Abstract

**Background:**

Effective treatment monitoring and treatment decisions in relapsing-remitting multiple sclerosis (RRMS) require accurate and individualized prediction of future disease courses. Guidelines from the Magnetic Resonance Imaging in Multiple Sclerosis (MAGNIMS) group and the Canadian Multiple Sclerosis Working Group (CMSWG) frequently cite MRI outcomes as predictive, but the methodological quality of this evidence is uncertain.

**Objectives:**

This study aims to critically assess the methodological standards underlying predictive claims about MRI outcomes in four major relevant MS guidelines.

**Design:**

We conducted a content review of citations in the MAGNIMS 2015 and 2021 and the CMSWG 2013 and 2020 guideline publications.

**Methods:**

Each source was evaluated for whether it reported quantitative predictive evidence: either predictive values with confidence intervals, Kaplan–Meier–based risk estimates, or externally validated models that provide accurate risk estimates (good calibration) and correctly separate high- from low-risk patients (good discrimination); We also checked if measures such as correlations, odds ratios, hazard ratios, Prentice criteria, or likelihood ratio tests were used.

**Results:**

Across all four guidelines, most predictive statements relied on secondary citations and association-based measures. Odds ratios, hazard ratios, correlations, or Prentice criteria were commonly reported. Some studies reported predictive values, but confidence intervals were frequently not provided. Only isolated examples of properly validated prediction models were cited, and only one had undergone full external validation. Advanced methods, such as the likelihood reduction factor, were absent.

**Conclusion:**

Current guideline statements on MRI prediction in RRMS often rely on associations rather than validated individualized predictions. They do not quantify individual risk or provide evidence for accuracy, calibration, discrimination, or robustness (reliability of predictions across different patients and settings). To ensure trustworthy and actionable evidence, future guidelines should require prospective risk estimates with confidence intervals, externally validated models with calibration and discrimination, predefined thresholds for predictive usefulness, and evaluation of clinical utility (e.g., decision curve analysis).

## Plain Language Summary

Why was the study done? To effectively treat relapsing-remitting multiple sclerosis (RRMS), reliable individualized prediction of disease worsening based on MRI findings is needed. Four widely used guidelines cite sources that provide such predictive information. However, it is unclear if the presented evidence supports good individual predictions. Thus, this study aims to assess the quality of predictive information in the literature cited by the guidelines on the prediction quality of MRI in RRMS. What did the researchers do? The sources claiming predictive ability of MRI in two guidelines from the Magnetic Resonance Imaging in Multiple Sclerosis (MAGNIMS) group and two guidelines from the Canadian Multiple Sclerosis Working Group (CMSWG) were extracted. The objectives, methodology, and content of the sources were analyzed. The methodologies were then grouped into ten statistical categories, and each category was assessed for its quality in individual prediction. What did the researchers find? In total, 75 sources were identified, which were directly or indirectly cited in the guidelines to show the predictive quality of MRI information. About 80% of sources used association measures to show individual prediction. The cited evidence was mostly insufficient to enable clinically relevant individual predictions. Neither groups report the quality of evidence they used in their guidelines. They also do not report measures for uncertainty of estimates (e.g., confidence intervals). However, one study included an independently tested model, while one other study used a statistically sound prediction model. What do these findings mean? Current guideline statements on MRI prediction in RRMS often rely on associations and rarely employ well-validated methods. There is a need for the Multiple Sclerosis scientific community to set minimum standards for the evidence accepted to support individualized prediction and to rank and assess the contribution of each evidence.

## Background

Personalized (or precision) medicine aims to tailor prevention, diagnosis, and treatment strategies to individual patients rather than applying “one-size-fits-all” approaches. This requires understanding which patients are likely to benefit or be harmed by a particular treatment, based on their molecular, imaging, or clinical profiles. Prediction models estimate the probability of a future clinical outcome (e.g., treatment response, relapse, survival) based on patient-specific features. They can be prognostic (predict individual disease course regardless of treatment) or predictive (estimate differential treatment benefit or harm). Prediction models make personalized medicine operational, but only through shared decision-making (SDM) do individualized predictions translate into care choices aligned with patient values.

**Figure 1.**
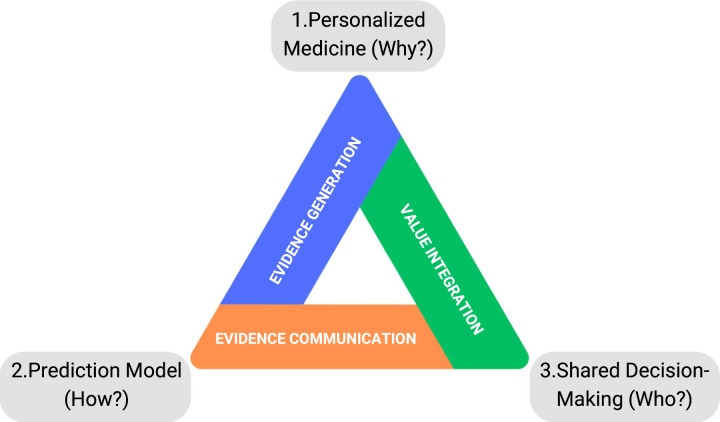
The triangular relationship between “Personalized Medicine ”, “Prediction Model” and “Shared-Decision Making”.

There is a triangular relationship between 1) Personalized Medicine (“why?” or “under what circumstances?”), 2) prediction models (“how?” or “how decisions are made?”), and 3) the shared decision making (“who?” or “who participated in the decision?”) as shown in Figure 1. The quality of prediction models is the keystone of the personalized medicine and shared decision-making triangle: only when models are well-validated and clinically useful can personalization be trustworthy and decisions be genuinely shared.

If prediction models are valid and robust, the flow from data → personalization → shared decisions is coherent. When methodologically inappropriate models provide individual prediction, both personalization and shared decision-making are compromised: the triangle collapses into opinion-based medicine rather than evidence-based personalization.

The treatment of multiple sclerosis (MS) involves a multifaceted approach to personalized medicine and encompasses various types of immunotherapy with different mechanisms of action, routes of administration, and risk profiles.^1^ New or enlarging T2 lesions despite immunotherapy indicate a treatment failure. If a breakthrough disease cannot be ruled out and the patient is adhering to treatment, the option is to escalate the therapy. This can prevent further relapses and delay disability by ensuring treatment is optimized on time.^2-6^

Treatment response monitoring uses prognostic models for the future disease course based on valid response biomarkers. Early treatment failure detection and consequent treatment switches or escalation enable optimal individual management in relapsing-remitting multiple sclerosis (RRMS). The MAGNIMS (Magnetic Resonance Imaging in MS) group and CMSWG (Canadian Multiple Sclerosis Working Group) guidelines have made important contributions to the treatment and management of MS based on MRI biomarkers.

The MAGNIMS guidelines^5,6^ provide standardized MRI protocols and criteria for diagnosis, prognosis, and monitoring disease activity, which are integral for SDM. For example, a patient on a disease-modifying treatment (DMT) who shows new T2 lesions despite no clinical relapse may be identified as having radiological disease activity, prompting a switch to a more effective treatment option.

CMSWG also focuses on optimizing the clinical utility of MRI in MS.^2,3^ This provision of guidance is conducive to the consistent utilization of MRI for the timely detection of disease progression. This ensures that any necessary alterations in treatment strategy can be implemented on time. The guidance encourages the use of imaging evidence to facilitate SDM.

A survey among US neurologists revealed that 97% of physicians adhere to the recommendation of monitoring relapsing-remitting MS (RRMS) within 12 months by MRI. Furthermore, when two or more new T2 lesions occur, 67% of patients undergo a treatment switch.^7^ While neither MAGNIMS nor CMSWG issues direct treatment algorithms, they underpin the SDM process in RRMS by defining how MRI is used.

MAGNIMS and CMSWG define practices that lead physicians to recommend treatment changes to their patients following an unfavorable MRI assessment.^2,5,6^ However, systematic reviews on prediction models in MS^8,9^ concluded that most prediction models are inadequately validated and not yet recommended for clinical use, highlighting the need for the MS field to develop a more nuanced understanding of the elements that constitute a high-quality individual prediction.

Both guidelines enhanced interpretability by standardizing definitions and acquisition protocols. They improved the clarity and consistency of MRI findings, thereby facilitating the process by which clinicians and algorithms can make accurate predictions. Predictions can be made sooner, thus improving opportunities for early intervention. Timeliness is enhanced by ensuring early and regular imaging, while relevance is augmented by the direct correlation of MRI findings with treatment and prognosis in RMS. This ensures that MRI-based predictions are clinically meaningful.

However, in addition to its practical utility, a high-quality prediction must also be statistically sound. The focus of the present paper is on the evidence on prediction quality in the MAGNIMS and CMSWG guidelines. To achieve this objective, we perform a qualitative content analysis, collect, critically assess, and classify the methodologies employed by both guidelines and their updates when assessing evidence for the predictive potential of MRI-based measures. Our goal is to discuss contributions to a methodological roadmap for future guideline development.

Despite the argument that guidelines are not designed as methodological gold standards, but as pragmatic tools, we examine the MAGNIMS and CMSWG guidelines with a methodological and biostatistical focus. Medical guidelines sit in a tension between methodological rigor and clinical practicality: if they privilege one side, the “personalized medicine–prediction model–shared decision-making” triangle tilts, but when both are balanced, guidelines become the bridge that makes robust methodology usable in everyday patient care. Balanced guidelines should explicitly state model quality criteria (validation, calibration, clinical utility) and provide tools for communication in practice (risk calculators, patient decision aids). The following review elucidates the context of prediction in both guidelines. Only when guidelines combine methodological rigor with clinical practicality do prediction models serve as a stable foundation for personalized medicine and shared decision-making in the treatment of RRMS patients.

## Methods

A qualitative content analysis (QCA)^10^ was performed on all articles to which both guidelines and their extensions refer, as well as the papers in the generations before (chains of citation). It is a structured yet interpretive method of analyzing textual material, aiming to uncover meanings, patterns, and themes. In this specific case, it critically assesses the methodology used to claim evidence for prognostic statements. For more details, see Supplementary Text 1. The methodological strategies were then classified based on four criteria (accuracy, discrimination, calibration, robustness) outlined in Table 1.

**Table 1.** Evidence for Predictive Validity Provided by Specific Biostatistical Methods

Method	What it provides	Accuracy (A)	Calibration (C)	Discrimination (D)	Robustness (R)	Comment
Group comparisons (responders vs. non-responders)	Retrospective contrasts of MRI outcomes	✗	✗	✗	✗	No individual risk quantification
Sensitivity & Specificity	Conditional probabilities of MRI outcome given disease outcome	✗	✗	✗	✗	Do not translate into individual risk
Odds Ratios/Hazard Ratios	Relative measures of association	✗	✗	✗	✗	Require baseline risk to inform prediction
Prentice criteria/PTE	Association & mediation at population level	✗	✗	✗	✗	Insensitive to individual variability
Likelihood Ratio Test (LRT)	Compares model fit with/without MRI	✗	✗	✗	✗	Significance depends on sample size
Predictive Values (PPV, NPV)	Risk within MRI-defined groups	✓	✓	✓	✓	Valid if CIs reported
Kaplan–Meier curves	Time-dependent risk estimates	✓	✓	✓	✓	CIs quantify precision of estimates
Correlation/r^2^	Shared variance between MRI & outcome	✓	✗	✓	✓	Thresholds needed to define good prediction
LRF/Mutual Information	Variance reduction in complex models	✓	✗	✓	✓	Not used in guidelines; thresholds required
Prediction models (validated)	Externally validated AUC + calibration	✓	✓	✓	✓	Gold standard

✗ - No, ✓- Yes

The literature was screened by two authors (DM and UM), and the results were discussed and harmonized by three authors (DM, SB, and UM). Medical issues were discussed with our clinical partner (JH). For each reference in the guidelines, a search was conducted for the paper containing the original results, and the chain of cross-references was recorded. A detailed content analysis of the predictive statements made is provided in the supplement.

The QCA defines a prediction rule as “high-quality” if it is accurate, reliable, and actionable. **Accuracy** refers to the closeness of the prediction to the true occurrence of the future event. It is assessed by the degree to which predicted probabilities correspond to observed frequencies (**calibration**) and by the ability of the model to distinguish between different outcomes (**discrimination**).^11^ Reliability reflects the **robustness **and generalizability of the model, i.e., its ability to perform consistently across different settings, including an external validation on independent data.^12^ Actionability emphasizes that predictions must support practical SDM. This requires that predictions are interpretable, fostering user trust and understanding of the rationale behind the model outputs.^13^ Predictions must also be timely and relevant, meaning that they are delivered early enough to inform decisions and address the clinical question within its proper context.^14^

The following paragraphs explain how we judge the specific methodology that we found in the material studied:

A **significant difference in MRI outcomes between response groups** is sometimes interpreted as predictive. However, such retrospective contrasts cannot quantify the predictive value of MRI and do not establish individual risk or provide evidence for accuracy, calibration, discrimination, or robustness (A, C, D, R). For example, Tomassini and colleagues,^15^ cited in three guidelines, compared baseline MRI between responders and suboptimal responders after six years.

**Sensitivity** (probability of an unfavorable MRI outcome among patients with unfavorable clinical outcomes) and **specificity** (probability of a favorable MRI outcome among patients with favorable outcomes) are also cited as predictive, but they likewise fail to establish individual risk or provide evidence for A, C, D, R. Durelli et al.,^16^ cited in MAGNIMS 2015 and 2020, reported sensitivity, specificity for MRI activity and neutralizing antibody (Nab) positivity at six months in relation to worsening at 18 months.

**Odds ratios** (OR) and **hazard ratios** (HR) are relative measures. Without baseline risk (the probability of an event when all risk factors are assumed yielding their pre-defined baseline values), they cannot quantify individual prediction or provide evidence for A, C, D, R. Río et al.,^17^ cited in three guidelines, reported a high OR (8.3, 95% CI 3.1–21.9) for MRI at 12 months predicting disability progression at two years.

The **Prentice criteria** test whether treatment affects both clinical and MRI outcomes, whether MRI is associated with clinical outcome, and whether MRI mediates a treatment effect^18^(Prentice, 1989). From these, the proportion of treatment effect explained (PTE) can be derived using estimates of appropriate regression models.^19^ However, these regression-based estimates summarize effects for the entire population group and do not tell us how well the treatment or MRI predicts outcomes for an individual patient. Thus, they provide no evidence for A, C, D, R. An example is Sormani et al.,^20^ cited in both MAGNIMS guidelines, which linked T2 lesion number and relapse rate utilizing PTE. Supplementary Text 2 illustrates how PTE remains equal to 1 regardless of individual variability, again failing to support individual prediction.

The **likelihood ratio test** (LRT) compares model fit with and without MRI outcomes. A significant result only shows that including MRI improves model fit, not that it improves individual prediction, especially in large samples. Thus, LRT does not provide evidence for A, C, D, R. Prosperini et al.,^21^ cited in all four guidelines, used LRT and Cox models to identify predictors of EDSS worsening.

**Predictive values** (positive predictive value, PPV; negative predictive value, NPV) quantify risks within MRI-defined groups and, when accompanied by confidence intervals, provide evidence for A, C, D, R. Prosperini et al.^22^ reported PPVs and NPVs with CIs to define lesion thresholds predictive of worsening during four years.

**Kaplan–Meier curves** estimate time-dependent risks within MRI-defined groups. When risk estimates and CIs are reported, they provide evidence for A, C, D, R. For example, Sormani et al.^23^ compared disability progression over time between baseline MRI groups, though without Cis and using the same data as for model development (no external or internal validation).

**Correlation coefficients** (r) and **coefficients of determination** (r^2^) are frequently reported. A significant correlation alone does not imply predictive utility. Thresholds for r^2^ and lower confidence bounds above those thresholds are needed to claim precise prediction. This provides evidence for A, D, R, but not calibration. Rudick et al.,^24^ cited in CMSWG 2020, reported only Pearson correlations between T2 lesions and disease severity. The figure in Supplementary Text 2 provides insight into how correlation relates to the precision of prediction.

**The likelihood reduction factor** (LRF) and **mutual information** (MI) extend r^2^ to more complex models. Both quantify reduction in variability, with higher values indicating stronger prediction. Evidence for A, D, R is possible if thresholds are defined, but no guideline cites this method.

Finally, **prediction models** are methodologically sound only if discrimination (by the area under the curve - AUC) and calibration (intercept and slope) are externally validated. Internal AUCs alone are insufficient, because a model can seem accurate on the original data yet provide systematically incorrect risk estimates for new patients, resulting in miscalibration and poor generalizability. Properly validated models provide evidence for A, C, D, R. For example, Rudick et al.^25^ selected models based on AUC.

## Results

### MAGNIMS

In MAGNIMS 2015,^5^ we identified four predictive statements on the role of MRI outcomes for future disease course (Supplementary Figure 1). Most references were secondary citations rather than original studies. Supplementary Tables 1–2 summarize methodological details. Among cited references, five reported predictive values, one reported a prediction model with missing external validation, seven provided Prentice criteria or PTE values, seven used odds or hazard ratios without full model specification, and four cited correlations without thresholds for clinically relevant precision.

In MAGNIMS 2021,^6^ five predictive statements were identified (Supplementary Figure 2; Tables 3–4). Again, most references cited secondary sources. Six reported predictive values, two Prentice criteria or PTE, six odds or hazard ratios without full model specification, and one correlation without thresholds.

### CMSWG

The 2013 edition^2^ contained six predictive statements (Supplementary Figure 3; Tables 5–6). Sources again relied on secondary citations. Two papers presented prediction models with no external validation, four reported predictive values, seven cited odds or hazard ratios without reporting the full models or baseline risks, and two provided correlations without thresholds.

In the 2020 update,^3^ four predictive statements were identified (Supplementary Figure 4; Tables 7–8). Eleven sources reported predictive values, one Prentice criteria/PTE, eleven odds or hazard ratios without models or baseline risks, and two correlations without thresholds.

In summary, across all four guidelines, predictive statements on MRI outcomes were frequent but largely based on secondary citations. Most sources reported relative measures such as odds ratios, hazard ratios. Some sources reported correlations or Prentice criteria (PTE). Only a minority presented predictive values, and a few described prediction models, generally without external validation or calibration. None of the guidelines cited advanced measures such as the LRF. An overview is provided in Table 2.

**Table 2.** Synopsis of Methodology Across the Guideline Documents

Methodology	MAGNIMS 2015	MAGNIMS 2021	CWGMS 2013	CWGMS 2020
Group comparison	M: 6 S: 0	M: 6 S: 0	M: 7 S: 0	M: 7 S: 0
Sensitivity, Specificity	M: 2 S: 0	M: 4 S: 0	M: 3 S: 0	M: 4 S: 0
OR, HR	M: 7 S: 0	M: 6 S: 0	M: 7 S: 0	M: 11 S: 0
Prentice criteria, PTE	M: 7	M: 2	M: 0	M: 1
Likelihood Ratio Test (LRT)	M: 1	M: 2	M: 1	M: 1
Predictive values	S: 3	S: 4	S: 3	S: 6
Subgroup-specific Kaplan-Meier curves	S: 2	S: 2	S: 1	S: 5
Correlation	M: 4 S: 0	M: 1 S: 0	M: 2 S: 0	M: 2 S: 0
Likelihood reduction factor (LRF)	M: 0 S: 0	M: 0 S: 0	M: 0 S: 0	M: 0 S: 0
Prediction models	M: 1 S: 0	M: 0 S: 0	M: 2 S: 0	M: 0 S: 0
Percentage of sound methods	M: 28 S: 5 15.2%	M: 21 S: 6 22.2%	M: 22 S: 4 15.4%	M: 26 S: 11 29.7%

S: Number of sources using a methodology that can provide reliable information on prediction quality (sound).

M: Number of sources using a methodology that cannot provide reliable information on prediction quality (misleading).

Most information provided does not allow to quantify patient-related risks.

## Discussion

Clinically actionable prediction in RRMS requires transparent risk statements with uncertainty and prediction horizon, not association metrics. Statements like *“At 12 months after treatment initiation, two new T2 lesions are observed; the two-year risk of disease activity or EDSS progression is 72% (95% CI 67–77%).”* meet this bar when such risk estimates are based on calibrated, discriminating, and decision-ready models and pair a point estimate with quantified uncertainty and a clear prediction horizon.

Such statements can be obtained via: (i) predictive values when both MRI and outcome are dichotomized, with 95% CIs; (ii) Kaplan–Meier analyses that estimate time-dependent risks within MRI-defined strata, allowing horizon-specific predictive values and CIs; and (iii) well-calibrated, externally validated prediction models that report individual risks with calibration (intercept, slope) and discrimination (AUC).^11,26^

Rudick et al.^25^ provided a seminal paper that explored definitions of treatment response to interferon-β in MS by classifying patients according to relapse counts and on-treatment MRI activity. Their analysis showed that subgroups with high numbers of new MRI lesions during therapy exhibited greater clinical and structural progression, whereas relapse-based definitions lacked specificity. Importantly, baseline characteristics could not reliably predict response status, highlighting the limitations of pre-treatment prognostication. While this work illustrates the potential of dynamic, on-treatment markers to refine response classification, it remains essentially a subgrouping exercise rather than a validated individual-level prediction model.

Efthimiou et al.^27^ present a guidance paper on the appropriate methodology for developing and validating predictions of RRMS disease progression. The paper provides a 13-step guide. The authors develop a Bayesian logistic mixed-effects prediction and test its calibration via a calibration curve and its discrimination via the area under the curve (AUC). Furthermore, the clinical utility of the model is assessed using decision curve analysis, and suitable thresholds are provided.

Frequently, studies assess predictive potential rather than deliver individualized risks, using R^2^ (coefficient of determination) or its generalization, the likelihood reduction factor (LRF).^28,29^ Both quantify uncertainty reduction by regression models. Additionally, LRF is suitable for logistic, Poisson, or survival models: for example, an RRMS logistic model including new T2 lesions, treatment, age, and EDSS, yielding LRF = 0.27 (95% CI 0.23–0.32). This number needs a qualitative interpretation, which can be seen as a modest information gain relative to an uninformative model, which does not consider any patient-related characteristics.

To judge clinical relevance, thresholds for R^2^/LRF are needed. They can help to decide whether it’s worth developing a full predictive model. RRMS studies rarely prespecify them. As a cross-domain reference, IQWiG^30^ considers LRF < 0.50 clinically irrelevant in oncology. Establishing RRMS-specific thresholds is a priority for future research.

**Figure 2.**
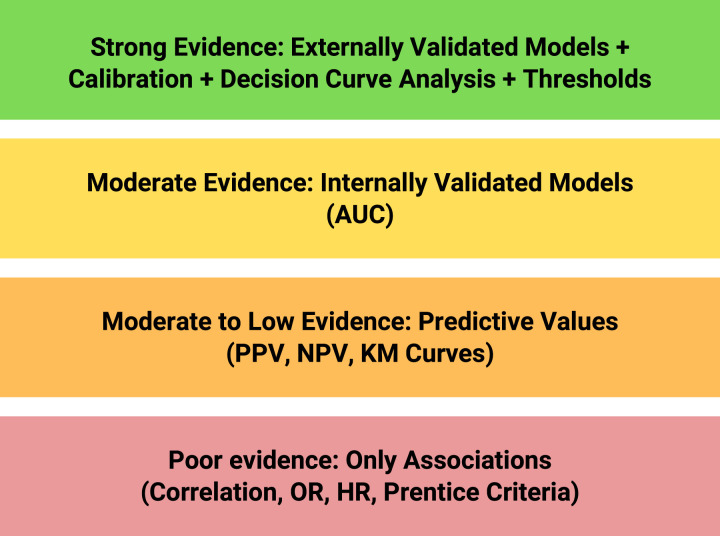
The hierarchy of statistical evidence for individualized prediction in relapsing-remitting multiple sclerosis.

Our review also highlights the frequent reliance on methods that do not support individualised prediction, such as the Prentice criteria or PTE. Future guidelines should prioritise evidence that meets the standards of accuracy, calibration, discrimination and robustness, drawing on recent best-practice papers such as those outlined by Efthimiou et al.^27^ Figure 2 shows the evidence hierarchy.

Guideline development should pivot from association-centric evidence towards risk-centric, validated predictions. Guidelines should report PPV/NPV (with CIs) or horizon-specific KM-based risks, and adopt externally validated models with calibration metrics. Based on our discussions, we propose a methodological roadmap for future guideline development, presented in Supplementary Text 3.

To close the loop to practice, guidelines should also incorporate decision curve analysis to quantify the net benefit of treatment for individual patients across relevant disease-progression risk-thresholds, demonstrating whether using a prediction model actually improves treatment decisions compared with treating all or none.^31-33^ Recent methodological primers^27^ offer a template for this shift.

Finally, our focus on four guidelines and on statistical criteria is a limitation; nonetheless, the pattern is consistent: association is abundant; individualized, calibrated risk is scarce. Redirecting evidence standards toward A, C, D, R (see Table 1) will make MRI-based prediction trustworthy and actionable for SDM.

## Conclusion

Our review shows that guideline statements from MAGNIMS and CMSWG regarding the predictive value of MRI in RRMS frequently depend on association-based methods, such as correlations, odds ratios, hazard ratios, or Prentice criteria, which are unable to quantify the quality of individual predictions. In contrast, approaches such as predictive values with confidence intervals, subgroup-specific Kaplan–Meier curves, the mutual information-based LRF or externally validated models with calibration and discrimination are the exception rather than the rule.

This imbalance is important because prediction forms the basis of individualized, evidence-based shared decision-making (SDM) in MS care. MRI remains clinically valuable, but the way predictive evidence is currently assessed and reported should be adapted in the future to meet the requirements of modern predictive research.

Future guidelines should therefore set minimum requirements for predictive claims:

(1) Report prospective risk estimates (e.g., predictive values or survival-based estimates) alongside confidence intervals.
(2) Ensure that prediction models are externally validated with calibration and discrimination metrics.
(3) Define thresholds for predictive usefulness (e.g., minimal R^2^ or LRF values).
(4) Evaluate clinical utility with decision curve analysis.

Adopting such standards, similar to initiatives like TRIPOD,^34^ would greatly improve the interpretability, timeliness and relevance of MRI-based predictions in RRMS and provide a stronger basis for clinical guidelines.

Although MRI remains central to RRMS care, only risk estimates with confidence intervals, validated models and demonstrated clinical utility can provide trustworthy predictions for individualised treatment decisions.

## Supplemental Material

Supplemental Material - Correlation Is Not Prediction: Reassessing Predictive MRI Evidence in Guidelines for Persons With Relapsing-Remitting Multiple Sclerosis Supplemental Material for Correlation Is Not Prediction: Reassessing Predictive MRI Evidence in Guidelines for Persons With Relapsing-Remitting Multiple Sclerosis by Dulat Minas, Stefan Buchka, Joachim Havla and Ulrich Mansmann in Journal of Central Nervous System Disease

## Data Availability

All data are presented in the electronic supplement.*
