# Supplementary material for: Correlation Is Not Prediction: Reassessing Predictive MRI Evidence in Guidelines for Persons With Relapsing-Remitting Multiple Sclerosis
Source: J Cent Nerv Syst Dis. 2026 Jun 25;18:11795735261453967. doi: 10.1177/11795735261453967 (PMC13305512; doi:10.1177/11795735261453967)
Supplement: Supplemental Material - Correlation Is Not Prediction: Reassessing Predictive MRI Evidence in Guidelines for Persons With Relapsing-Remitting Multiple Sclerosis [file sj-pdf-1-cns-10.1177_11795735261453967.pdf]

Supplementary Material

Supplementary Text 1. Qualitative Content Analysis

Supplementary Text 2. Commenting on the predictive irrelevance of PTE (Proportion of Treatment Effect explained)

Supplementary Text 3. Minimal Reporting Checklist for Predictive Claims in MS Guidelines

Supplementary Table 1. Assessment of the References in MAGNIMS 2015

Supplementary Figure 1. References Used in References of MAGNIMS 2015

Supplementary Table 2 Classifying the Evidence Strategies Used to Claim Predictive Value of MRI in MAGNIMS 2015

Supplementary Table 3. Assessment of the References in MAGNIMS 2021

Supplementary Figure 2. References Used in References of MAGNIMS 2021

Supplementary Table 4. Classifying the Evidence Strategies Used to Claim Predictive Value of MRI in MAGNIMS 2021

Supplementary Table 5. Assessment of the References in CMSWG 2013

Supplementary Figure 3. References Used in references of CMSWG 2013

Supplementary Table 6. Classifying the Evidence Strategies Used to Claim Predictive Value of MRI in CMSWG 2013

Supplementary Table 7 Assessment of the References in CMSWG 2020

Supplementary Figure 4. References used in References of CMSWG 2020

Supplementary Table 8 Classifying the Evidence Strategies Used to Claim Predictive Value of MRI in CMSWG 2020

Supplementary References

Supplementary Text 1: Qualitative Content Analysis

The Qualitative Content Analysis (QCA) was performed according to the procedures outlined by Mayring (2015). QCA is a systematic method for analyzing textual material (in our case, journal articles) to interpret meanings, patterns, and themes within the data. Unlike purely quantitative content analysis, which counts word frequencies, categories, or co-occurrences, qualitative content analysis focuses on interpreting the content—that is, both what is explicitly stated and the underlying meanings.

Supplementary Text 1: Figure 1. Flowchart of the QCA methodology

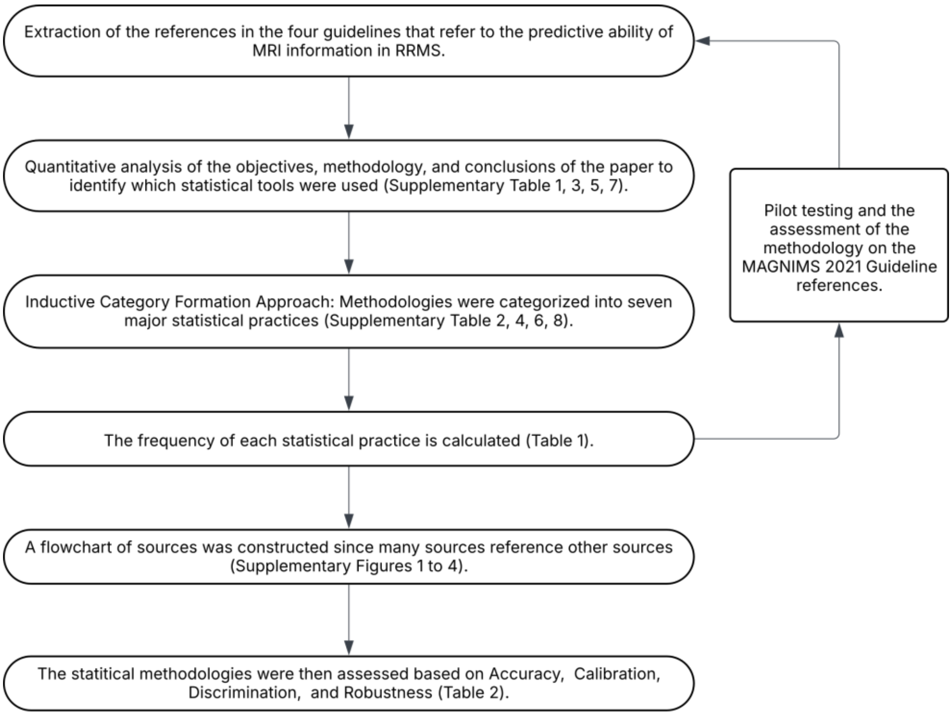

Key Features:

- Mixed Method: QCA combines quantitative analysis, which is a frequency analysis in this research, and qualitative analysis, which is qualitative-interpretive analysis with a hermeneutical logic in assigning categories. This means that papers were critically examined beyond just the statistical aspect and included the objectives and conclusions of the paper.
- Systematic and rule-guided: Follows a structured process (coding, categorization, theme development) to ensure rigor and transparency as shown above. Pilot testing of the methodology on one guideline is performed.
- Inter-coder reliability: Two authors have carried out the methodology, with a further check from the third author.
- Inductive Category Formation Approach that does not allow a biased pre-defined set of statistical methodologies.

Supplementary Text 2: Commenting on the predictive irrelevance of PTE (Proportion of Treatment Effect explained)

This short section explains why the PTE (proportion of treatment effect explained) is not an appropriate measure to quantify the quality of individual prediction. The PTE is derived from the regression models which underly the Prentice criteria.

Please have a look at the following figure;

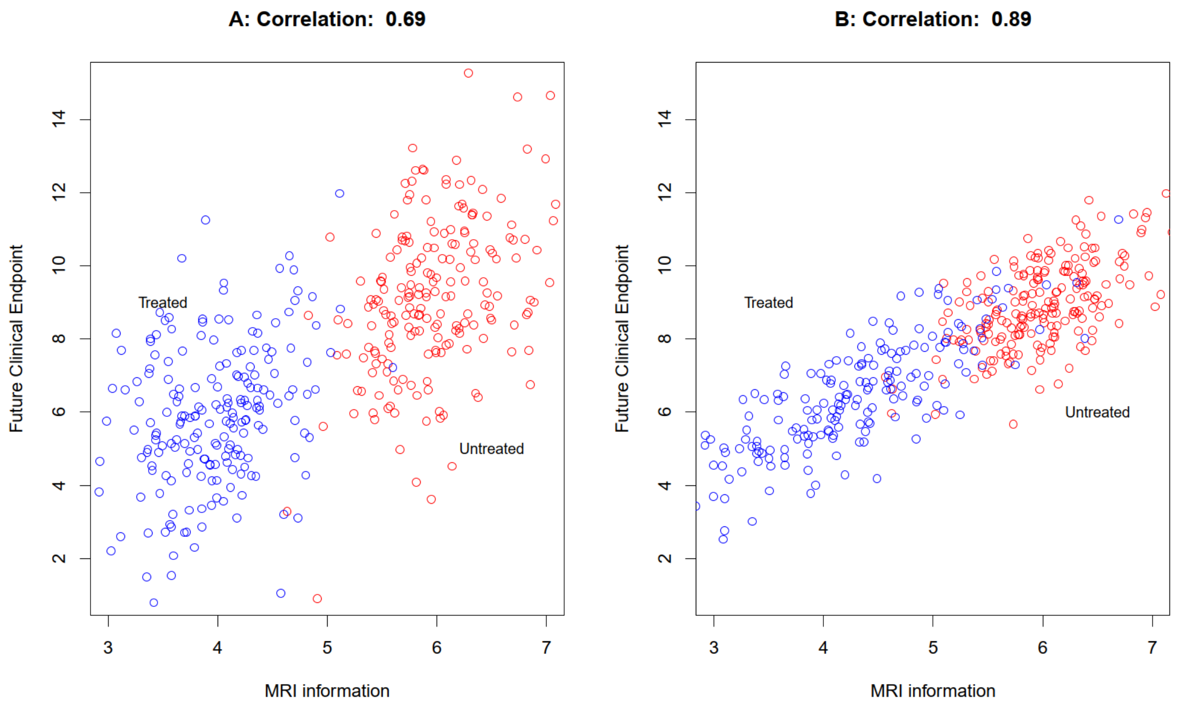

Both panels (A and B) show artificial settings were the MRI measurement (x-Axis) is associated with a respective clinical outcome (y-axis). The red dots belong to untreated while the blue dots belong to treated subjects. The treatment reduces the MRI measure in mean from 6 to 4 as well as the clinical outcome in mean from 9 to 6. The treatment effects on both outcomes (MRI and clinical) are the same in both panels. The formal regression models are defined as follows:

Model 1: MRI ~ Treatment
Model 2: CE ~ Treatment
Model 3: CE ~ SE
Model 4: CE ~ Treatment + MRI

The PTE is calculated as (T.1 – T.4)/T.1, where T.1 is the estimated treatment effect in Model 1 and T.4 is the estimated treatment effect in Model 4. In the given example, the treatment effect in Model 4 is 0 because the MRI measurement explains the clinical outcome to a full extent. It is a perfect surrogate. Therefore, in both panels, the PTE is equal to 1.

But it is obvious that the prediction of the clinical outcome given the MRI information is more precise in panel B, with a correlation (r) of nearly 0.9 compared to panel A, with a correlation (r) of about 0.7. In terms of the coefficients of determination (r^2), we have 0.49 in Panel A and 0.81 in Panel B.

The practical problem is: When is the correlation large enough to guarantee a sufficiently good prediction? As mentioned in our text, the IQWIG uses a threshold of 0.5 for the coefficient of determination (the squared correlation).

Supplementary Text 3: Minimal Reporting Checklist for Predictive Claims in MS Guidelines

Purpose: To ensure that MRI-based predictive claims in relapsing–remitting multiple sclerosis (RRMS) are based on sound, reproducible, and clinically relevant evidence.

1. Study Design and Data

Prospective design (retrospective comparisons are insufficient for prediction quality).
Clearly defined population, MRI outcome, and clinical endpoint.
Report time horizon of prediction (e.g., 1-year, 2-year risk).

2. Predictive Performance

Report predictive values (PPV, NPV) with 95% confidence intervals.
Provide time-dependent estimates (Kaplan–Meier subgroup curves or survival-based predictions).
If models are used, report discrimination (AUC) and calibration (intercept, slope, calibration plots).

3. Model Validation

Perform external validation (not only internal cross-validation).
State whether the model generalizes across populations (robustness).

4. Quantification of Prediction Quality

Report r^2, LRF, or MI values with confidence intervals.
Define a threshold for clinical usefulness (e.g., r^2 or LRF $\geq$ predefined cut-off).
Avoid equating statistical significance (p-values for OR, HR, correlation) with prediction quality.

5. Clinical Utility

Assess net clinical benefit using decision curve analysis (DCA) or comparable methods.
Explicitly state how the prediction would change treatment decisions.

6. Transparency

Provide sufficient detail (or code/data, if possible) for reproducibility.
Declare limitations (statistical, clinical, or biological).

Key Principle: A predictive claim should only be made if the evidence includes calibration, external validation, and clinical utility assessment.

Supplementary Table 1. Assessment of the References in MAGNIMS 2015[2]

Claim made in MAGNIMS 2015	Original guideline citation (given in bold numbers) and as listed in the Supplementary References (superscripts)
**Claim 1:** MRI monitoring of disease course<br><br>Several guidelines have tried to define the indications for and frequency of serial MRI in adults and children with an established diagnosis of MS **(18–20)**.	**Claim in the Guideline 18[3]:** "T2-hyperintense lesions predict MS (second clinical attack) over short and long intervals, and change in T2 BOD [Burden of Disease] predicts long-term disability in populations (19)".<br><br>**Our Assessment:** T2 Burden of Disease is claimed to be a predictor for CEP, without providing information on the quality of individual risk estimates based on this predictor.<br><br>Source 19[4] establish T2 MRI activity with potentially predictive characteristic: "The EDSS score at 14 years [after diagnosis] correlated moderately with lesion volume on MRI at 5 years (r=0.60) [CI: 0.41-0.74] [after diagnosis] and with the increase in lesion volume over the first 5 years (r=0.61) [CI: 0.42-0.75]". This source employs Spearman correlation between MRI lesion volume and EDSS Score in CIS patients. Correlation is not a perfect tool to qualify prediction and surrogacy. It does not offer insight into individual risk estimates and their quality.
	**Claim in the Guideline 19[5]:** "Until recently, attempts to show that conventional MRI is a surrogate for clinical relapses and progression were limited and not very robust (76–82). This situation started to change when it was shown that MRI reflected not only pathology but also clinical prognosis and response to therapy. Subsequently, a meta-analysis of all large, randomized, placebo-controlled studies of patients with RRMS was carried out by Sormani et al. (106–109)... These and two subsequent studies based on individual patient analysis showed that MRI markers satisfy the rigorous Prentice criteria for consideration as a valid surrogate marker in group studies of RRMS (108–110)."<br><br>**Our assessment:** The paper does not make a clear statement about individual risk estimates and their quality. It provides qualitative evidence on potential surrogacy by using Prentice criteria (108-110) and the Coefficient of Determination from Meta-Analytic Weighted Regression Analysis (106-107).<br><br>Paper 106[6] assesses MRI lesions on a trial level, not an individual level, using a meta-analytic approach: weighted regression with coefficient of determination. They find that "strong correlation was found between the effect on the relapses and the effect on MRI activity" with "the adjusted R^2 value of the weighted regression line was 0.81". It does not provide information on individual risk estimates and their quality.<br><br>In Source 107[7], "A set of 19 randomized double-blind controlled trials in RRMS were identified, for a total of 44 arms, 25 contrasts, and 10,009 patients… correlation between the treatment effect on MRI lesions and EDSS worsening was slightly weaker (R(2) = 0.57) but significant" using a meta-analytic approach.<br><br>Source 108[8] uses the Prentice Criteria and PTE: "to evaluate surrogacy of 1-year MRI active lesions and relapses for disability worsening [EDSS] over the 2-year follow-up." The paper reports "1-year MRI lesion activity … accounted for more than 60% of the treatment effect on 2-year EDSS worsening [Proportion of Treatment Explained (PTE) = 63 (20-215)]. A combination of 1-year MRI lesion activity and

Claim made in MAGNIMS 2015	Original guideline citation (given in bold numbers) and as listed in the Supplementary References (superscripts)
	relapses explained 100% [PTE = 100 (32-311)]". A combination of 1-year MRI lesion activity and relapses [PTE = 100(32-311)]". The PTEs are not able to provide information on the quality of individual risk estimates.<br><br>In Source 109[9] "Individual-patient data from two large, placebo-controlled clinical trials of subcutaneous interferon β-1a in patients with relapsing–remitting or secondary progressive (SP) MS were analysed separately and as pooled data. The four Prentice criteria were applied to assess surrogacy for the number of new T2 MRI lesions. PTE on relapses … by the effect of treatment on new T2 MRI lesions over 2 years was 53% (28-101) in PwRRMS". This paper studies surrogacy in terms of the Prentice criteria in SPMS patients. They are helpful to establish a relationship between the SEP and the CEP on a group level, but they do not inform about the quality of individual risk estimates between SEP and CEP.<br><br>Source 110[10] is a methodology.
	**Claim in the Guideline 20[11]:** "The presence of contrast-enhancing lesions indicates new lesion formation, which is important in establishing an MS diagnosis according to dissemination in time (2,3). Along with markers of disease activity, such as annualized relapse rate and formation of new T2 lesions, contrast enhancement is also used as a surrogate marker of therapeutic efficacy in trials of MS treatment."<br><br>**Our Assessment:** This guideline mainly discusses the diagnostic value of MRI imaging, not the treatment response aspect. It mentions the aspect of surrogacy without a clear assessment; no reference is provided to support this aspect.
**Claim 2.** Contrast-enhanced T1-weighted sequences are recommended to detect acute inflammation. However, depending on the clinical situation and the scan interval, demonstration of active (new or enlarging) T2 lesions can deliver sufficient information about subclinical disease activity and disease progression (Figure 1) (**24,25**).<br><br>T2-weighted and contrast-enhanced T1-weighted brain MRI are the modalities of choice for MS disease	**Reference 24[9]** has been discussed above in Supplementary Table 1 (Claim 1) as Source 109.
	**Claim in the Reference 25[12]:** While some studies (26–28) suggest a certain predictive value at the individual patient level, MRI markers and clinical outcomes are generally poorly correlated (29).<br><br>**Our Assessmen**t: This study acknowledges poor correlations, but also cites some evidence on the predictive value of the MRI marker on an individual level.<br><br>In Source 26[13], "a validation procedure was applied to clinical and MRI data collected in the context of a randomized, double-blind, placebo-controlled trial of glatiramer acetate in patients with relapsing-remitting MS. The four Prentice operational criteria were applied to assess surrogacy for the number of new enhancing lesions, the percentage change of T2 lesion volume, and a composite MRI score" on relapse rate.<br><br>Source 27[14] reports results for SPMS patients: T2-weighted MRI activity was validated as a surrogate for SPMS using Prentice Criteria. This statement does not provide evidence on individual prediction quality.

Claim made in MAGNIMS 2015	Original guideline citation (given in bold numbers) and as listed in the Supplementary References (superscripts)
monitoring, revealing acute and active inflammation, and clinically silent disease progression (**24,25**).	Source 28[9] has been discussed above in Supplementary Table 1 (Claim 1) as Source 109.<br><br>Source 29[15] uses the following methodological approach to test the predictive values of Contrast Enhancing Lesions (not T2 lesions) on relapse rate, relapse status, and time-to-first-relapse: "A predictive (regression) model for the relapse outcome was developed using a carefully guided stepwise forward selection procedure (with Poisson, logistic, and proportional hazards regression). Each step would examine whether the putative CEL surrogate outcome continued to add predictive capability beyond that provided by the included baseline covariates". The results are qualitative in terms of "improving prediction". The paper did not report any predictive relationship between MRI Imaging and CEP. The statistical analysis in phase 2 consisted of correlation analyses (Spearman rank and Pearson correlation coefficients with corresponding 95% confidence intervals) to assess the concurrent and predictive relationships between relapse outcomes and putative CEL surrogates.
**Claim 3.** Another approach to the prediction of treatment response is to analyse variables measured after the start of treatment, but before the actual clinical endpoint of interest. Several studies have attempted to define criteria and strategies for the early identification of suboptimal response in individual patients via a combination of clinical and MRI measures during the first 6–12 months after treatment initiation **(67,78–82)**. These criteria are partially or completely based on the detection of disease activity in follow-up brain MRI scans, defined as new gadolinium-enhancing lesions or new and/or enlarging T2 lesions compared with baseline scans.	**Claim in the Reference 67[16]:** "This is a prospective and longitudinal study of relapsing-remitting multiple sclerosis (RRMS) patients treated with IFNβ. Patients were classified based on the presence of new lesions on MRI, relapses, confirmed disability increase, or combinations of all these variables after 1 year of therapy. Regression analysis was performed in order to identify variables of response after a follow-up of 3 years". Results: "only the combination of new active lesions on MRI with the presence of relapses (OR 4.4; 95% CI 1.6-12.5) or disability progression (OR 7.1; 95% CI 1.6-33.9), or both (OR 6.5; 95% CI 1.9-23.4) achieved significant values to identify those patients with a poor outcome".<br><br>**Our Assessment**: OR does not provide direct information on individual risk predictions. Moreover, this study lacks a control group that is essential for ILS.
	**Reference 78[17] contains three claims. Additional claims from Reference 78[17] are also discussed in Supplementary Table 3 as Source 91.**<br><br>**Claim 1:** "As discussed above, both clinical relapses and active lesions on MRI have been shown to represent good surrogate markers of clinical disability in MS. Indeed, an analysis published in 2011 (53) showed that, according to the Prentice criteria for surrogate marker validation, (49) both 1-year MRI active lesions and clinical relapses independently accounted for more than 60% of the effects of IFN-β on 2-year EDSS worsening."<br><br>**Our Assessment**: The review cites Sources 53 and 49, which use the Prentice Criteria to assess surrogacy. They do not help assess the quality of individual risk estimates.<br><br>Source 49[10] is methodology and has already been mentioned in Supplementary Table 1.<br>Source 53[8] has been discussed above in Supplementary Table 1 (Claim 1) as Source 108.<br><br>**Claim 2:** "A recent summary of all the evidence indicates that, at the individual patient level, the effect of IFN-β on MRI active lesions

Claim made in MAGNIMS 2015	Original guideline citation (given in bold numbers) and as listed in the Supplementary References (superscripts)
	mediates more than the 60% of the effect on relapses, (52) while another study has shown that MRI lesions mediate about 57% of the IFN-β effect on disability progression. (51) The role of relapses as a valid surrogate for disability progression over a 2-year timeframe has been studied in IFN-βtreated (53) and natalizumab-treated (54) patients, in whom relapses accounted for 62% and over 80%, respectively, of the treatment effect on disability. Taken together, these observations provide a solid basis for considering MRI activity and relapses as potential markers of response to IFN-β, and they suggest that patients with MS who are likely to derive an IFN-β-induced benefit with regard to disability progression can be identified early by their MRI lesion and relapse activity."<br><br>**Our Assessment:** This claim reports the Proportion of Treatment Explained (PTE) to show that MRI markers are a good surrogate for disability progression. PTE is not sufficient for individual risk estimates. It also cites sources 53 and 54 to show that relapses are also a good surrogate for disability.<br><br>Source 51[14] has been discussed in above in Supplementary Table 1 (Claim 2) as Source 27.<br>Source 52[9] has been discussed above in Supplementary Table 1 (Claim 1) as Source 109.<br>Source 54[18] uses: 1) A hypothesis testing approach based on the Prentice Criteria, 2) the proportion of treatment effect explained, 3) an adjusted likelihood reduction factor, 4) the proportion of information gain. These methods are acceptable. However, in Source 54, only the short-term relapse activity is measured as a surrogate, not MRI data. This paper only offers more (3-4) or less (1-2) methodologically sound approaches to assess ILS. Thos source only assesses relapses and not MRI markers.<br><br>**Claim 3:** "By contrast, the development of one new T2 lesion greatly increased the risk of disability progression over 4 years in another study (29). Most studies did show that MRI lesion activity was associated with high relapse frequency over the follow-up period. (26,28–31)"<br><br>**Our Assessment**: The first sentence suggests that one new T2 lesion is a good predictor of the disability progression, while the second sentence merely mentions the association.<br><br>Source 26[19] "evaluated the predictivity of an active scan, NAb positivity, or both, during the first 6 months of treatment, on the occurrence of clinical disease activity in the following 18 months. Results: 147 patients were assessed at 16 centres. Active scan - sensitivity (SN) 52% (34–69%), specificity (SP) 80% (65–91%), negative predictive value (NPV) 73% (58–77%), positive predictive value (PPV) 62% (42–79%), p=0.002; … active scan and NAb positivity, SN 71% (38–91%), SP 86% (73–94%), NPV 94% (86–98%), PPV 50% (29–70%), p=0.0003". PPV for active scan includes 50%.<br><br>Source 28[20] reports ORs from a logistic regression analysis as a measure for the predictive ability of the SEP regarding the CEP: "active lesions in the scan performed at 12 months [after treatment start] were the most important factor related with the increase of disability after 2 years of therapy (odds ratio 8.3, 95%CI: 3.1–21.9; $p < 0.0001$)". The OR is an indirect measure. In case of a low incidence of the positive SEP it is also a relative risk. A large OR indicates that the risk under the unfavorable SEP is higher compared to the risk of the favorable SEP, but the risks are not quantified. PPV 37% (27-47) "of having more than two active lesions in the MRI performed at one year for

Claim made in MAGNIMS 2015	Original guideline citation (given in bold numbers) and as listed in the Supplementary References (superscripts)
	treatment response at 2 years”.<br><br>Source 29[21] states the risk of an unfavorable CEP given the unfavorable SEP after 1 year of INFB treatment. After “1 year of interferon beta (IFNB) therapy”, PPV and NPV of “Active MRI Scan” are 70.0 (49.5–71.6) and 77.6 (72.5–82.1) for poor outcome respectively. The PPV and NPV of “New T2-hyperintense lesions” for poor outcome are 71.1 (63.4–77.9) and 95.4 (91.9–97.7) respectively for poor outcome. The methodology is acceptable. However, this is only relevant information for PwRRMS under a specific treatment but does not describe general robust patterns for general PwRRMS populations. HR is also reported to be HR 16.8, 95%CI 7.6–37.1, P < 0.001 (new T2 lesions vs. being poor responder).<br><br>Source 30[22] reports HRs as indirect measures for a personal risk estimate: “The association between disability progression and risk group, as defined by the score, was evaluated by Kaplan Meier survival curves and Cox regression, and quantified by hazard ratios (HRs)”. A HR does not tell the true risks of the PwRRMS which can be read from the Kaplan Meier curves.<br><br>Reference 31[23] states that “To identify the predictors of disease progression and relapses, odds ratios, their corresponding 95 % confidence intervals (CIs), and p-values were computed using the logistic regression model”. This paper communicates OR as a surrogacy measure that also does not inform about the quality of individual risk predictions.
	**Reference 79**[22] is discussed above in Supplementary Table 1 as Source 30..
	**Claim of the Reference 80**[24]: This paper investigates the “four-year outcomes of interferon beta (IFNB)-treated patients with multiple sclerosis (MS) according to their clinical or magnetic resonance imaging (MRI) activity status at first year of treatment. They found that “An increased risk of relapses and disability worsening was found in patients … experiencing isolated MRI activity (HR = 3.15, and HR = 5.31) at first year of treatment, when compared with stable patients (all *p* values <0.001)”. “As the main time variable, we used the length of the observation (in years) between the end of first year of IFNB treatment, and the last follow-up visit, or the achievement of outcome”. *≥3 new T2-hyperintense lesions had a PPV of* 90% (80–96), NPV54%(48–60), SE 31% (24–37).<br><br>**Our assessment**: KM Curves are reported and support the findings, but with no CIs. This paper is methodologically acceptable, but the Sensitivity for T2 lesions is low.
	**Source 81**[25] is CMSWG 2013 Recommendations, which is reviewed separately in Supplementary Table 5.
	**Source 82**[26] **is a decision model that has three claims of interest.**<br><br>**Claim 1:** “MRI measures provide a greater sensitivity than relapses for MS-related disease activity [Barkhof et al. 1992]. A connection

Claim made in MAGNIMS 2015	Original guideline citation (given in bold numbers) and as listed in the Supplementary References (superscripts)
	between MS activity detectable by MRI and progression of impairment was described in multiple studies [Brex et al. 2002; Weiner et al. 2000].”<br><br>**Our Assessment:** The first sentence compares MRI measures to relapses, and sensitivity is not sufficient to identify a good predictor. The second sentence describes only the connection, not prognostic value.<br><br>[Barkhof et al. 1992][27] is a descriptive study on seven participants.<br>[Brex et al. 2002][4] has already been discussed in Source 18 in Supplementary Table 1 as Source 19<br>[Weiner et al. 2000][28] uses the repeated measures logistic regression models as a prediction measure on 45 well-defined untreated multiple sclerosis patients in different categories of disease (relapsing-remitting, progressive, stable),<br>which is not sufficient for ILS and treatment modification.<br><br>**Claim 2:** “Progression of the lesion load in the first year of an immunomodulatory therapy correlates with earlier progression of impairment. The probability of progression rose significantly by at least one EDSS point within the follow-up monitoring period of 4.8 years with the number of new T2 lesions after 1 year of therapy [Prosperini et al. 2009], namely from 5% (no new lesion) to 83% (at least three new lesions).”<br><br>**Our Assessment**: This claim slightly overstates the results of the paper. While 83% suggests a high risk of disability worsening, the formal positive predictive value (PPV) for any new lesions was 71% (95% CI: 63–78%).<br><br>[Prosperini et al. 2009][21] will be discussed in Source 66 in Supplementary Table 3 as Source e17.<br><br>**Claim 3: “**Rudick and colleagues found a significant correlation between the occurrence of more than two new T2 lesions during treatment with interferon β (IFNβ) and progression of impairment over the course of 2 years [Rudick et al. 2004]. Similarly, Rio and colleagues reported a higher risk of therapeutic failure during IFNβ treatment in patients who developed more than two active lesions (new or enlarging T2 lesions and new Gd-enhancing lesions) in the first year of therapy [Rio et al. 2008]. Pozzilli and colleagues observed a (s)lower EDSS progression over the course of 4 years in patients being treated with interferon β with absence of Gd-enhancing lesions as well as active T2 lesions in the first year [Pozzilli et al. 2005].”<br><br>**Our Assessment**: The first sentence of the claim states that the correlation was found, which does not necessarily indicate prediction and surrogacy. The second sentence compares two groups depending on the number of new MRI lesions. The last sentence cannot be supported, as the source cannot be found.<br><br>[Rudick et al. 2004][29] report: “regression model that included new T2 lesions, baseline T2 volume and change in gadolinium lesions during

Claim made in MAGNIMS 2015	Original guideline citation (given in bold numbers) and as listed in the Supplementary References (superscripts)
	the 2 years had the highest area under the ROC curve (0.69) of all models tested with three or less factors." This is an acceptable risk model development. There is no external validation of the model. They do not provide a calibration curve to understand the quality of individual risk estimations based on the proposed model.<br><br>[Rio et al. 2008] and [Pozzilli et al. 2005] were not listed in the reference list.<br>[Rio et al. 2008][20] will be discussed in Supplementary Table 3 as Source e15; [Pozzilli et al. 2005] will be discussed in Supplementary Table 5 as Source 71.
**Claim 4.** MRI should be included in drug surveillance programmes to screen for opportunistic infections, (103,114) unexpected disease activity (including paradoxical reactions), (82,115,116) and comorbidities (7,93)	**Source 82**[26] is discussed above as Source 82.
	**Source 115**[30] is a case report.
	**Source 116**[31] is a case report

Supplementary Figure 1. References Used in References of MAGNIMS 2015

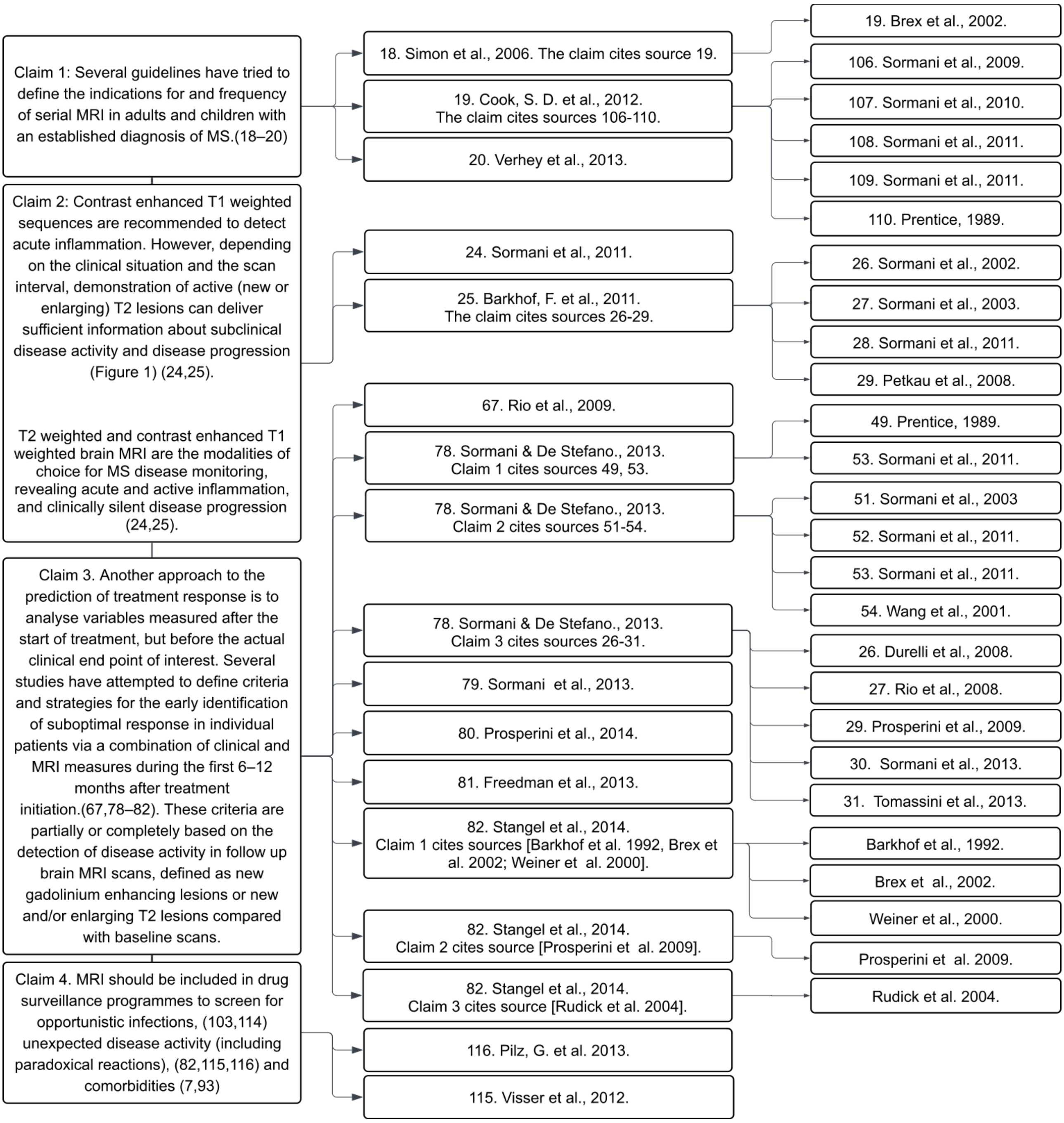

The diagram above shows the visual representation of citation flow in the MAGNIMS2015 Guideline used as evidence for predictive ability of MRI markers. The first column represents statements from the guideline and the references used therein. The second column represent sources of evidence as cited in the guideline (first line literature), the third column refers to papers cited in first line literature.

Supplementary Table 2. Classifying the Evidence Strategies Used to Claim Predictive Value of MRI in MAGNIMS 2015[2]

**Claim**	**Predic-tive Models**	**Group-specific predictive values (PPV, NPV, KM Curves as Indirect Information)**	**Sensitivity and Specificity**	**Prentice Criteria PTE**	**OR, HR without model reported**	**Correlation**	**Comparing groups**	**Likelihood Ratio Test (LRT)**	**Not aimed to measure the predictive quality of MRI**
MRI monitoring of disease course **…**				19[5][108[8],109[9]]	19[5][108[8]]	18[3][19[4]], 19[5][106[6], 107[7]]	18[3][19[4]]		20[11], 19[5][110[10]]
Contrast enhanced T1 weighted …				24[9], 25[12][26[13], 27[14], 28[9]]	25[12][29[15]]	25[12][29[15]]	25[12][29[15]]		
Another approach to the prediction of treatment response …	82[26] [Rudick[29]]	78[17][26[19], 28[20], 29[21]](PPV reported), 78[17][30[22]], 79[22](KM Curves reported), 80[24] (KM Curves and PPVs reported), 82[26][Prosperini[21]] (PPV reported),	78[17][26[19], 28[20], 29[21]]	78[17][49[10], 51[14], 52[9], 53[8], 54[18]]	78[17][28[20], 29[21], 30[22], 31[23]], 79[22], 82[26][Weiner[28], Prosperini[21], Rio[20]], 67[16], 80[24]	82[26][Brex[4]]	67[16], 80[24], 78[17][28[20], 29[21], 31[23]]	78[17][29[21]]	82[26] [Barkhof[27]]
Recommendations for levels of concern based …	82[26] [Rudick[29]]	82[26][Prosperini[21]] (PPV reported)	82[26][Prosperini[21]]		82[26][Weiner[28], Prosperini[21], Rio[20]]	82[26][Brex[4]]	82[26][Prosperini[21]]		82[26] [Barkhof[27]], 115[30], 116[31]

The table classifies the references discussed in Supplementary Table 1 according to methodological aspects. Numbers refer to the references in the guideline, superscripts to the Supplementary References. Numbers in square brackets represent the sources as cited in the first-line literature.

Supplementary Table 3. Assessment of the References in MAGNIMS 2021[32]

Claim made in MAGNIMS 2021	Original guideline citation (given in bold numbers) and as listed in the Supplementary References (superscripts)
**Claim 1:** Monitoring of treatment effectiveness and prediction of treatment response<br><br>The increasing number of approved disease-modifying treatments for relapsing multiple sclerosis, and also for primary progressive multiple sclerosis and secondary progressive multiple sclerosis with proven inflammatory disease activity, has further expanded the therapeutic landscape **(66).** This expansion further stresses the need for standardized MRI acquisition (i.e., reference and follow up scans) and reporting (appendix pp 4–6) to assess treatment effectiveness and predict treatment response **(67).**	**Claim in the paper *66*[33]**: “Ongoing disease activity, measured either by clinical relapses or new MRI-detected lesions (including unequivocally new T2 or new gadolinium-enhanced lesions), could lead to physical or cognitive worsening over time (e14−e17) … Such lack of response to a DMT has been difficult to define, as most people with MS are not free of all disease activity; investigators have considered using the number of clinical attacks or new MRI-detected lesions in the preceding 12 months to define lack of response (e15, e17).”<br><br>**Our Assessment**: In the above statement the authors define a surrogate called “no response” implying that no response has future consequences. Implicitly, they make a predictive statement: No response indicates an unfavorable disease course for the non-responding PwRRMS. They do not provide measures to quantify the quality of the prediction based on the defined surrogate. They reference sources which supposedly provide information on surrogate quality.<br><br>Source e14[34] states “In those developing multiple sclerosis, a concurrent correlation of change in T_2 lesion volume with change in EDSS was most evident in years 0–5 (r_s = 0.69, P< 0.001)”. The reported Spearman rank-correlation demonstrates a potential relationship between SEP and CEP. It does not quantify the quality of how the SEP is predicting the CEP.<br><br>Source e15[20] has been discussed in Source 78 in Supplementary Table 1 (Claim 3) as Source 28.<br>Source e16[22] has been discussed in Source 78 in Supplementary Table 1 (Claim 3) as Source 30.<br>Source e17[21] has been discussed in Source 78 in Supplementary Table 1 (Claim 3) as Source 29.
	**Claim in the Paper 67**[35]: “Post hoc analyses of clinical trials on IFN-β-1a and -1b clearly showed that the treatment effect on new T2 lesions in the first treatment year accounted for a relevant proportion of treatment effect on relapses (70%–80%) and disability worsening (60%) over the subsequent year. (24,26,27)”<br><br>**Our Assessment:** The statement formulates the risk of relapse or disability worsening in the following year given a new T2 lesion the year before and provides positive predictive values. Negative predictive values are missing. The paper quotes three source papers.<br><br>Source 24[8] has been discussed in in Source 19 in Supplementary Table1 (Claim 1) as Source 108.<br>In Source 26[9] has been discussed in in Source 19 in Supplementary Table1 (Claim 1) as Source 109.<br>Source 27[14] has been discussed in Source 25 in Supplementary Table 1 (Claim 2) as Source 27.
**Claim 2:** Extensive literature examines various prognostic scores for identifying treated patients with high risk of developing relapses and disability worsening; these studies were discussed in detail in the	**Claim in the Paper 2[2]:** This paper is MAGNIMS2015[2] and is assessed in Supplementary Table 1.
	**Claim in the Paper 84**[36]: “Treatment eff ects on ARR [Annulized Relapse Rate] were significantly higher in patients with than those without gadolinium activity (RE = 0.86 vs. RE = 1.15, P = 0.005)”. Methodological comment: RE is the ratio between subgroup HR and overall HR.

**Claim made in MAGNIMS 2021**	**Original guideline citation (given in bold numbers) and as listed in the Supplementary References (superscripts)**
previous guidelines **(2)** and are further supported by other studies **(84,85).** Models for the prediction of treatment response are mainly based on clinical and MRI measures that are collected one year after treatment onset, although one study showed the possibility to refine and personalise the prediction of treatment effect by use of pre-treatment demographic, clinical, and radiological characteristics **(86).**	**Our Assessment:** The statement describes a potential ILS between gadolinium activity as SEP and ARR as CEP. The reported result does not allow to quantify the individual risks of unfavorable CEP given individual gadolinium activity. No KM Curves were given.
	**Claim in the Paper 85**[37]: "≥3 new T2 lesions (HR: 2.9, 95% CI: 1.5–5.6); or ≥2 Gd-enhancing lesions (HR: 2.1, 95% CI: 1.1–4) were able to identify patients with EDSS worsening". The PPV for both ≥1 new and ≥4 T2 lesions was 25% "after 1 year of treatment for the different outcomes [≥2 point EDSS worsening] after 8 years of follow-up". The PPV is low with no CIs reported.<br>**Our Assessment:** While the HR is not directly addressing individual risks, the paper reports PPVs as well as NPVs but with no CIs.
	**Claim in the Paper 86**[38] is based on a multivariate post hoc analysis using baseline and outcome data of three RCTs (using methodology from Zhao et al. 2013[39]). It was found that the "The best response score defined on the ALLEGRO and the BRAVO was a linear combination of age, sex, previous relapses, brain volume, and MRI lesion activity. …in the ALLEGRO, the hazard ratio (HR) for disability progression of *laquinimod* vs placebo was 0.38 for responders, HR = 1.31 for non-responders (interaction $p = 0.0007$)". Similar results were obtained in BRAVO (HR = 0.40 for responders and HR = 1.24 for non-responders (interaction p = 0.006)) and successfully replicated in CONCERTO (HR = 0.44 for responders and HR=1.08 for non-responders (interaction p = 0.033)).<br><br>**Our Assessment**: The paper reports HRs and not individual risk estimates. It is not straightforward how to use this information for individual risk prediction. The paper provides evidence that the scores describe potential ILS.
**Claim 3:** A new baseline brain scan that is done more than a few months after treatment initiation is recommended for patients who are treated with disease-modifying treatments that require a long period of time to reach their full effect **(86,87)** (eg, glatiramer acetate, which takes up to 9 months to become effective) or with induction therapies, for which there is no value of obtaining a new baseline MRI scan until completion of the full initial course (figure 3) **(88,89)**.	**Claim in the Paper 87**[40]**:** "A number of studies have demonstrated a relationship between the development of new MRI lesions and a suboptimal clinical response to IFN- treatment. Among a cohort of more than 400 RRMS patients, the ones who developed new T2 lesions on MRI after one year of therapy had an increased risk of poor response to treatment (hazard ratio 16.8; P <0.001). The level of risk increased with the number of lesions observed independently of new relapses or disability progression [54, 55]".<br><br>**Our Assessment:** The presented results do not inform about the quality of individual predictions. The HR is an indirect measure on the change of a prediction under a changed baseline setting.<br><br>Paper [54][16] states: "Only those patients positive for MRI activity and relapses [OR 8.3 (2.9–28.9)] or for the three variables (MRI activity, relapses and progression) [OR 9.8 (2.6–53.4)] during the first months of therapy had a significant risk of developing new relapses in the following years". The authors provide a diagnostic OR which reflects Sensitivity and Specificity but does not help to derive predictive values. Aim was to investigate MRI and clinical predictors of response during the first 12 months of therapy. Logistic regression analyses were used to evaluate the association between predictor factors and response variables.<br><br>Paper [55][21] states the risk of an unfavorable CEP given the unfavorable SEP after 1 year of INFB treatment. After "1 year of interferon beta (IFNB) therapy", PPV and NPV of "Active MRI Scan" are 70.0 (49.5–71.6) and 77.6 (72.5–82.1) for poor outcome respectively. The PPV and NPV of "New T2-hyperintense lesions" are 71.1 (63.4–77.9) and 95.4 (91.9–97.7) respectively for poor outcome. The methodology is acceptable. However, this is relevant information for PwRRMS under a specific treatment but does not describe general robust patterns for general PwRRMS populations.

Claim made in MAGNIMS 2021	Original guideline citation (given in bold numbers) and as listed in the Supplementary References (superscripts)
	**Claim in the Paper 88**[41]: "The mean total number of enhancing lesions was 36.80 for the placebo group and 25.96 for the GA group (Fig 1). The mean reduction in the total number of enhancing lesions in the GA group compared with the placebo group was -10.8 (95% confidence interval [CI], -18.0 to -3.7; p=0.003), a 29% reduction". The methodology: "A baseline-adjusted analysis of covariance (ANCOVA) compared the two study arms for the primary end point, incorporating terms for treatment and center as main effects". A simple comparison of two groups is not a good prediction model.<br><br>**Our Assessment**: A simple comparison of two groups is not a good prediction model. Moreover, the paper acknowledges that "The uncertain relationship between frequency of enhancement and accumulation of irreversible disability (20) also does not allow us to comment on whether the ability of the drug to suppress inflammation is one of the fundamental mechanisms through which GA has shown to reduce the accumulation of disability in patients with RRMS".
	**Claim in the paper 89**[42]**:** The aim of the paper was to prove **"**the effectiveness of alemtuzumab in reducing the relapse rate and accumulation of disability compared with IFNα-1a through extended follow-up (up to 60 months from baseline)**"**<br><br>**Our Assessment:** This paper does not report on the predictive value or ILS ability of MRI lesions.
**Claim 4**: In the absence of a new baseline scan, gadolinium-enhanced T1-weighted sequences done 3–6 months after treatment onset can also be helpful to identify ongoing activity because interval active T2 lesions might be related not to ineffective treatment but to the therapeutic lag of the drug during the first few months of therapy (panel 4) **(90).**	**Claim in the Review 90**[43]**:** "Conventional MR imaging measures, such as T2 lesion volume/number, do not fully correlate with clinical measures of disability in patients with MS, but there is growing evidence that MR imaging at the onset of the disease may serve as a prognostic marker of disability accumulation (17). A recent study showed that the number of enhancing lesions in patients treated with IFN-b was the best predictor of disability increase measured by the Expanded Disability Status Scale (EDSS) (50).<br><br>**Our Assessment:** This paper acknowledges that T2 lesion activities are not fully predictive for CEP, but cites and presents sources that have different findings.<br><br>Source 17[44] reports that "patients … displaying more than 10 brain T2 lesions were associated with a worse outcome (HR 4.3; 95% CI 2.4–8.0)" compared to the patients with 0 lesions. They report an HR (indirect measure for change of risks) and not individual risk estimates and their quality. KM Curves are also reported.<br>Paper 50[45] is discussed in Supplementary Table 7 as Source 98.
**Claim 5:** …in patients with suspected clinical activity that is not confirmed on brain or spinal cord MRI, a new brain MRI scan 6 months later can be considered. In these situations, the persistence of clinical or radiological disease is sufficient to identify patients with	**Claim in the Paper 91**[17]: "A recent summary of all the evidence indicates that, at the individual patient level, the effect of IFN-β on MRI active lesions mediates more than the 60% of the effect on relapses (52), while another study has shown that MRI lesions mediate about 57% of the IFN-β effect on disability progression (51)".<br><br>**Our Assessment:** It is a group statement about the change of a specific aggregated risk between two treatment groups. No information on individual risk. Sources 51 and 52 are already cited in Source 67 in Table 1.<br><br>Source 51[14] has been discussed in Source 25 in Supplementary Table 1 (Claim 2) as Source 27.

Claim made in MAGNIMS 2021	Original guideline citation (given in bold numbers) and as listed in the Supplementary References (superscripts)
suboptimal treatment response **(91)**.	Source 52[9] has been discussed in in Source 19 in Supplementary Table1 (Claim 1) as Source 109.

Supplementary Figure 2. References Used in References of MAGNIMS 2021

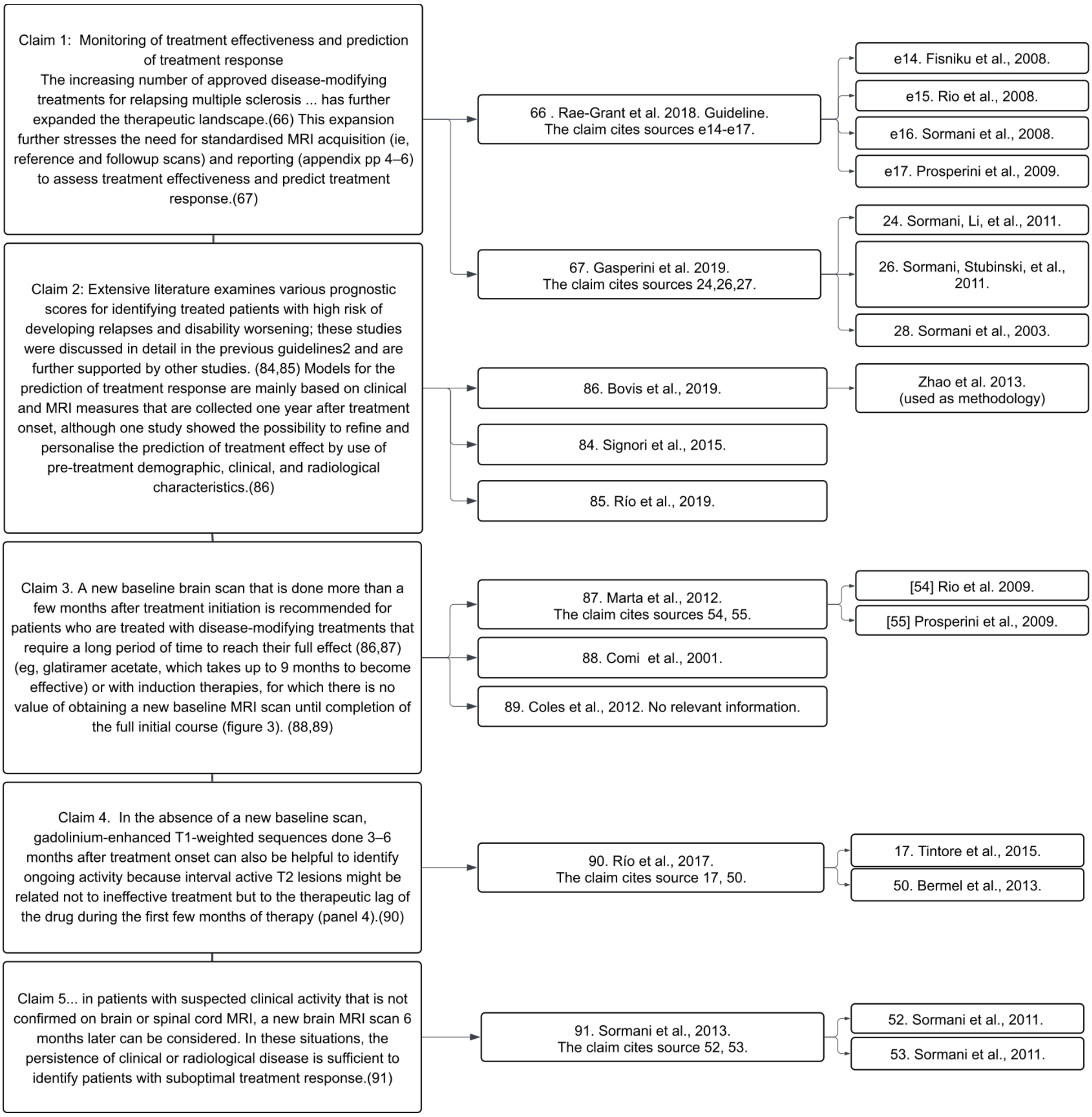

The diagram above shows the visual representation of citation flow in the MAGNIMS2021 Guideline used as evidence for predictive ability of MRI markers. The first column represents statements from the guideline and the references used therein. The second column represent sources of evidence as cited in the guideline (first line literature), the third column refers to papers cited in first line literature.

Supplementary Table 4. Classifying the Evidence Strategies Used to Claim Predictive Value of MRI in MAGNIMS 2021[32]

Claim	Predictive Models	Group-specific predictive values (PPV, NPV, KM Curves as Indirect Information)	Sensitivity and Specificity	Prentice Criteria PTE	OR, HR without model reported	Correlation	Comparing groups	Likelihood Ratio Test (LRT)	Not aimed to measure the predictive quality of MRI
Monitoring of treatment …		66[33][e16[22]] (KM curves reported), 66[33] [e15[20], e17[21]] (PPV reported)	66[33][e15[20], e17[21]]	67[35][24[8], 26[9], 27[14]]	66[33][e15[20], e16[22], e17[21]]	66[33][e14[34]]	66[33] [e15[20]], 66[33] [e17[21]]	66[33] [e17[21]]	
Extensive literature …		85[37] (PPVs reported with no CIs)	85[37]		84[36], 85[37], 86[38]		84[36], 86[38]		
A new baseline brain scan …		87[40][55[21]]	87[40][55[21]]		87[40][54[16]]		87[40][54[16]], 88[41]	87[40][55[21]]	88[41], 89[42]
In the absence of …		90[43][17][44] (KM curves reported) 90[43] [50[45]] (PPV reported with no CIs)	90[43][50[45]]		90[43][17[44]], 90[43][50[45]]		90[43][17[44], 50[45]]		
In patients with …				91[17][51[14], 52[9]]					

The table classifies the references discussed in Supplementary Table 3 according to methodological aspects. Numbers refer to the references in the guideline, superscripts to the Supplementary References. Numbers in square brackets represent the sources as cited in the first-line literature.

Supplementary Table 5 Assessment of the References in CMSWG 2013[25]

Claims made in CMSWG 2013	Original guideline citation (given in bold numbers) and as listed in the Supplementary References (superscripts)
**Claim 1.** Rio et al reassessed the clinical usefulness of different treatment failure criteria and found that a combination of either relapse or EDSS progression criteria together with MRI changes were sensitive at one year to predict continued activity should a patient remain on the same therapy **(66).**	**Source 66[46] makes four claims, which we will discuss one by one.**<br><br>**Claim 1:** "Clinical trials have shown that disease-modifying agents can have effects that are detectable on MRI. Notably, decreases in the T2-lesion load and in the number of gadolinium-enhancing lesions have been observed in some patients during the first few years of treatment (6–8)."<br><br>**Our Assessment:** The statement summarizes results from three papers.<br><br>Source 6[47] uses the ANOVA of ranks method to prove correlation. Correlation is not enough to inform about the quality of individual risk estimates. Moreover, this paper does not aim to establish MRI as a good predictor, rather it is aiming to support the efficacy of the drug.<br>Source 7[48] uses Spearman rank statistics and forward stepwise regression to identify characteristics that are predictive of the number of new, enlarging, and new plus enlarging lesions at year 2. This is not the issue of our research: We are interested in the predictive value of T2 lesion information on later clinical disease worsening.<br>Source 8[49] uses an ANOVA model on the ranks and reports the percent median increase or decrease for the Burden of Disease. The results are not informative on the quality of individual risk predictions. This paper does not aim to establish MRI as a good predictor, rather it is aiming to support the efficacy of the drug.<br><br>**Claim 2:** "Various studies have demonstrated that the presence of new disease activity on MRI in the first 2 years of treatment with iFn-β showed a marked correlation with the clinical response. Moreover, in clinical trials, treatment-induced changes observed on MRI enabled the treatment effect on relapse rate to be reliably predicted. (46,52)"<br><br>**Our Assessment:** The first part of the claim indicates correlation, which does not equal prediction. In the second part, reliable prediction by MRI of relapses suggests that there is good proof of the surrogacy of MRI activity.<br><br>Source 46[29] has already been discussed in Source 82 in Supplementary Table 1 (Claim 3) as [Rudick et al., 2004].<br>Source 52[6] has already been discussed in Source 19 in Supplementary Table 1 (Claim 1) as Source 106.<br><br>**Claim 3**: "The results from one study revealed that patients treated with disease-modifying agents who developed more than two new T2 lesions after 2 years of treatment had a raised risk of a poor treatment response (46). Another study found that the risk of a poor response, defined by an increase in disability after 2 years of treatment, was higher in those patients with three or more new active lesions after 12 months of treatment than patients with two or fewer active lesions (odds ratio 8.3, P<0.0001; Figure 1) (53)."<br><br>**Our Assessment:** The first part of the claim is the comparison of two groups to show the prediction of MRI markers, while the second part reports ORs, which are not sufficient to show a reliable predictive tool.<br><br>Source 46[29] has already been discussed in Source 82 in Supplementary Table 1 (Claim 3) as [Rudick et al., 2004].

Claims made in CMSWG 2013	Original guideline citation (given in bold numbers) and as listed in the Supplementary References (superscripts)
	Source 53[20] has already been discussed in Source 78 in Supplementary Table 1 (Claim 3) as Source 28.<br><br>**Claim 4**: "By assessing both the presence of enhancing lesions on a baseline MRI and the number of relapses over the previous 2 years, investigators were able to accurately estimate the short-term risk of relapse in patients with RRMS (55). Another study showed that the presence of at least of two of the three clinical or mri variables (relapses, an increase of disability, and new lesions on mri) during the first year of therapy enabled patients with a marked risk (odds ratio 5.9–13.2) of clinical activity within the ensuing 2 years to be identified (56)."<br><br>**Our Assessment:** The first part of the claim indicates a predictive characteristic of baseline MRI, while the second part reports ORs to show that MRI identified those at high risk. ORs are not sufficient to show prediction, although it may indicate a useful relationship.<br><br>Source 55[50] validates MRI activity (Gd-enhancing lesions) as an independent predictor of relapse over 9–12 months. In a Cox model, both lesion count (HR = 1.03, $p < 0.001$) and prior 2-year relapses predicted relapse risk. Patients with high MRI lesion burden had up to 74% relapse risk at 9 months**.** While MRI is confirmed as predictive, the paper provides a formula, not a clinical tool, for estimating risk.<br>Source 56[20] has already been discussed in Source 78 in Supplementary Table 1 (Claim 3) as Source 28.
**Claim 2.** The significance of MRI lesions in comparison to relapses is often questioned. In a randomized, placebo-controlled clinical trial of IFNβ-1a therapy for RRMS, **Rudick et al.** found that ≥2 Gd-enhancing lesions on MRI over two years were two-fold more predictive of EDSS progression than relapses; ≥3 or more new T2 lesions were 3.4 times more predictive than relapses **(70).** Thus, the evidence does not support the presumption that relapses are more important than MRI lesions as predictors of suboptimal response to therapy.	**Source 70**[29] has already been discussed in Source 82 in Supplementary Table 1 (Claim 3) as [Rudick et al., 2004].

Claims made in CMSWG 2013	Original guideline citation (given in bold numbers) and as listed in the Supplementary References (superscripts)
**Claim 3.** A number of studies have attempted to define poor responders to therapy and to identify standard MRI features that are predictive of poor response. In patients with RRMS, ≥1 new gadolinium-enhancing lesions or new T2 lesions after one year on therapy with IFNβ is associated with an approximate three to eight times higher risk of having ≥2 or more relapses (suboptimal treatment response) over the next four to five years **(71,72)**. In the study by **Tomassini et al (72),** the presence of Gd-enhancement or black holes at baseline was also associated with higher relapse rate (Gd-enhancement) and disability progression (black holes) on treatment over the next five years **(72).**	**Claim of the paper 71**[51]**: "**The presence of T1- enhancing lesions and new T2 hyperintense lesions on the scan performed after the first year of therapy were the best MRI features associated with both the occurrence of relapses during the treatment period (OR for enhancing lesions and relapses 3.6 (CI 95% 1.3–10.2); OR for new T2 lesion and relapses 2.8 (CI 95% 1.1–7.3))**".**<br><br>**Our Assessment:** The paper reports odds ratios that show MRI activity at 1 year is linked to a higher risk of future relapses. However, the study does not provide a practical tool or score to estimate relapse risk for individual patients. The findings help show a general trend, but they cannot be directly used in clinical practice to calculate a specific patient's future risk.
	**Source 72**[23] has already been discussed in Source 78 in Supplementary Table 3 as Source 31.
**Claim 4.** In a follow-up of two to three years, **Prosperini and colleagues** assessed the risk of EDSS progression associated with the presence of new T2 lesions on a scan one year after initiating therapy **(73).** They found that the risk of EDSS progression was increased about 10-fold for a single new T2 lesion, about 20-fold for two new T2 lesions, and about 30-fold for ≥3 new T2 lesions. Similarly, Rio and colleagues reported an approximate	**Source 73**[21] has been discussed in Source 78 in Supplementary Table 1 (Claim 3) as Source 29
	**Source 74**[20] has been discussed in Source 78 in Supplementary Table 1 (Claim 3) as Source 28.
	**Claim of the Paper 75**[52]**:** "We are interested in whether the inclusion of an indicator of patients meeting the threshold increases the predictive value of a model that already incorporates other predictive characteristics. To investigate this, we first fit a logistic model that relates the probability of relapse within 28 days of a visit to potential predictors… The model incorporating the predictive covariates is then expanded to include a patient-specific random effect and the threshold guideline is added. A significant adjusted odds ratio (OR) associated

Claims made in CMSWG 2013	Original guideline citation (given in bold numbers) and as listed in the Supplementary References (superscripts)
10-fold increase in the risk of progression over two years for patients with active lesions on MRI obtained one year after initiating therapy **(74).** An increase of ≥5 Gd-enhancing lesions in serial scans of a patient is associated with a significantly increased risk of a relapse within the next month **(75).**	with the guideline indicates that the guideline accounts for a portion of the risk of relapse that is unexplained by other factors."<br><br>**Our Assessment**: While the study demonstrates that MRI lesion activity improves short-term relapse prediction (28 days) when added to a clinical model, it does not evaluate or establish MRI as a surrogate marker for long-term disability progression, such as sustained EDSS worsening or irreversible functional decline in RRMS.
**Claim 5.** Recently, **Sormani et al** have analysed the Prevention of Relapses and disability by Interferon beta-1a Subcutaneously in Multiple Sclerosis **(PRISMS 76)** dataset to determine the predictive value of a combined metric of new T2 lesions and relapses at one year after treatment initiation as a surrogate for disability progression **(37).** Patients were assigned a modified Rio score based on new T2 lesions (>5=1 point) and relapses (1 relapse=1 point; >2 relapses=2 points). Scores ranged from 0 (new T2 lesions < 5, no relapses) to 3 (new T2 >5, >2 relapses). The risk of disease progression at three years (four years after starting treatment) for patients considered to be treatment responders (score 0 or 1) was 32-42%, and for treatment non-responders (score 2 or 3) was 50%. Thus, the combination of MRI lesions and relapses after treatment initiation appears to be a reasonable	**Source 37**[22] has been discussed in Source 78 in Supplementary Table 1 (Claim 3) as Source 30.<br><br>**Source 76**[53] is not intended to assess the predictive ability of MRI lesions. It is about the treatment effects of a drug. But it describes an important data set which can be used to study ILS between MRI information and later clinical disease course.

Claims made in CMSWG 2013	Original guideline citation (given in bold numbers) and as listed in the Supplementary References (superscripts)
surrogate of disease progression.	
**Claim 6.** Recommendations for levels of concern based on MRI are shown in Table 3. In keeping with the trend in recent guidelines **(10).** These recommendations expand the role of MRI in determining the level of concern regarding a suboptimal response to therapy compared to the previous CMSWG recommendations. The basic concept underlying these recommendations is that ongoing focal white-matter inflammation in the setting of a therapy that is supposed to modulate this inflammation (particularly IFNβ) is an indicator of suboptimal treatment response **(77).**	**Source 10**[54] does not mention MRI activity as a marker for treatment response or as a predictor of the poor outcome in RRMS. The focus of the paper is the diagnostic criteria.
	**Source 77**[3] **has already been discussed in Table 2 as Source 18.**

Supplementary Figure 3. References Used in References of CMSWG 2013

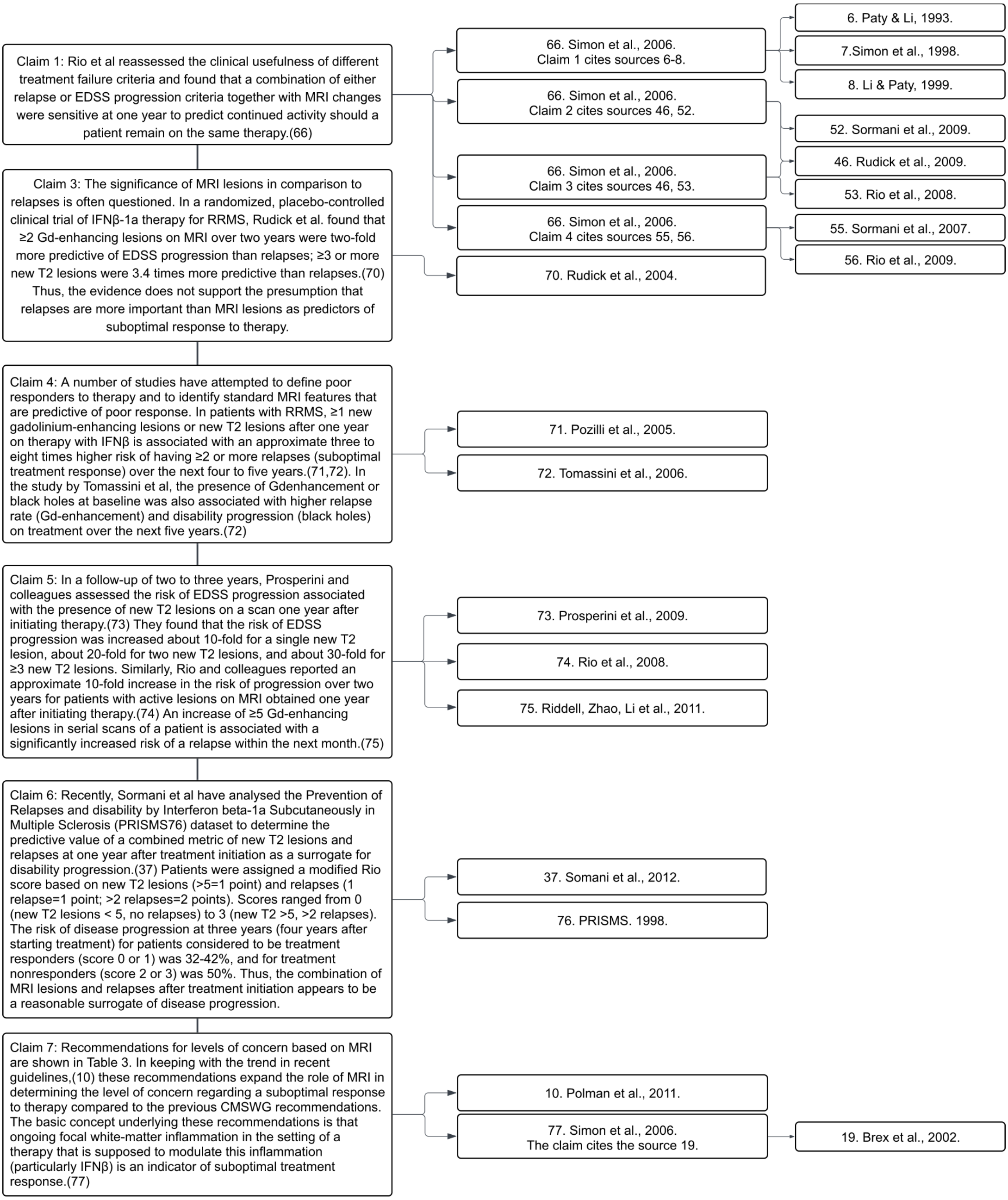

The diagram above shows the visual representation of citation flow in the CMSWG 2013 Guideline used as evidence for the predictive ability of MRI markers. The first column represents statements from the guideline and the references used therein. The second column represents sources of evidence as cited in the guideline (first-line literature), and the third column refers to papers cited in the first-line literature.

Supplementary Table 6. Classifying the Evidence Strategies Used to Claim Predictive Value of MRI in CMSWG 2013[25]

**Claim**	**Predictive Models**	**Group-specific predictive values (PPV, NPV, KM Curves as Indirect Information)**	**Sensitivity and Specificity**	**Prentice Criteria PTE**	**OR, HR without model reported**	**Correlation**	**Comparing groups**	**Likelihood Ratio Test (LRT)**	**Not aimed to measure the predictive quality of MRI**
Rio et al reassessed the clinical…	66[46][46[29]], 66[46][55[50]]	66[46][46[29]] (ROC Curve[a] reported), 66[3][53[20]](PPV reported)	66[46]		66[46][56[16]]		66[46][56[16]]		66[46] [52[6]], 66[46][6[47], 7[48], 8[49]]
The significance of MRI lesions in …	70[29]								
A number of studies have …					*72*[23], 71[51]		*72*[23], 71[51]		
In a follow-up of two to three years …		74[20](PPV reported), 73[21](PPV reported)	74[20], 73[21]		73[21], 74[20], 75[52]	75[52]	73[21], 74[20], 75[52]	73[21]	
Recently, *Sormani et al* have analysed…		37[22](KM Curves reported)			37[22]				76[53]
Recommendations for levels of concern …						77[3][19[4]]	77[3][19[4]]		10[54]

The table classifies the references discussed in Supplementary Table 5 according to methodological aspects. Numbers refer to the references in the guideline, superscripts to the Supplementary References. Numbers in square brackets represent the sources as cited in the first-line literature.
[a]ROC: Receiver Operating Characteristic Curve

Supplementary Table 7. Assessment of the References in CMSWG 2020[55]

**Claim made in CMSWG 2020**	**Original guideline citation (given in bold numbers) and as listed in the eReference (superscripts)**
**Claim 1.** Several CSF or serum biomarkers of disease activity and treatment response have been proposed but have not been sufficiently validated for use in clinical practice. In consequence, clinicians must infer a treatment response according to the level of disease activity, as demonstrated by relapses and/or active MRI lesions, after a treatment has been initiated. The rationale for this approach is the finding that ongoing relapses and active lesions over the short term are predictive of disability worsening in patients treated with interferon-β, teriflunomide, and fingolimod **(82-85).**	**Source 82**[8] has been discussed in in Source 19 in Supplementary Table1 (Claim 1) as Source 108.
	**Claim in Paper 83**[56]**:** "The association of 1-year MRI or clinical relapses with the risk of treatment failure (defined as EDSS worsening or treatment switch for inefficacy) and of EDSS worsening alone was evaluated using multivariate Cox models". Results: "The risk of failure had a relevant increase with … ≥3 new T2 lesions (HR 1.55, 95% CI 0.92–2.60, p=0.09). In patients without relapses and less than 3 new T2 lesions, the 3- year risk of failure and EDSS worsening were 17% and 15%; in patients with 1 relapse or ≥3 new T2 lesions, the risks were 27% and 22%; in patients with both conditions or more than 1 relapse, the risks were 48% (p =0.001) and 29% (p = 0.001)". PPV is also communicated: "risk levels were grouped in 3 classes: group 0 = those without relapses and less than 3 new T2 lesions; group 1=those with 1 relapse or ≥3 new T2 lesions; group 2 = those with 1 relapse and ≥3 new T2 lesions or ≥2 relapses.", where "Score 0 vs scores 1 or 2 had a PPV of 26% and an NPV of 86%, a sensitivity of 50%, a specificity of 70%, and a global accuracy of 66%"<br><br>**Our Assessment**: Cox Proportional Hazards with HRs are insufficient to characterize the quality of individual risk estimates. This paper also includes PPVs and NPVs. But no confidence intervals are reported, which would have elucidated the quality of the risk estimate.
	**Claim in the Paper 84**[57]: As a methodology, "a post hoc analysis was conducted in a subgroup of patients who received teriflunomide in the core study, had MRI and clinical relapse assessments at months 12 (n = 5 552) and 18, and entered the extension. Patients were allocated risk scores for disability worsening (DW) after 1 year of teriflunomide treatment: 0=low risk; 1=intermediate risk; and 2–3 = high risk, based on the occurrence of relapses (0 to ≥2) and/or active (new and enlarging) T2-weighted (T2w) lesions". Results: "In patients with a score of 2–3, the risk of 12-week–confirmed DW over 7 years was significantly higher vs those with a score of 0 (hazard ratio [HR] = 1.96, p =0.0044). Patients reclassified as high risk at month 18 (18.6%) had a significantly higher risk of DW vs those in the low-risk group (81.4% [were reclassified as low risk ("responders")]; HR = 1.92; p = 0.0004)".<br><br>**Our Assessment**: HRs are indirect measures for change of risk but do not express directly individual risk estimates. These may be read from Kaplan-Meier curves together with their confidence intervals. CIs for KM curves are not provided in this paper.
	**Claim in the Paper**[58]**:** Methodology: "Unadjusted logistic regression was used to assess whether focal MRI activity and/or ≥1 confirmed relapses during M0–M12 of treatment, could predict the likelihood of the following clinical events during M12–M24 and M12–M48". Results: "For disease activity during the first year, either focal MRI activity or ≥1 relapse were strongly and significantly predictive of relapses or 6M-CDP, while the combination of these disease activity measures was associated with the highest odds of relapses or 6M-CDP"<br><br>**Our Assessment**: This is a conference abstract, where only the poster and abstract version of this paper was found. The study uses logistic regression and reports ORs. The paper does not report individual risk estimates or strategies on how to derive them.

Claim made in CMSWG 2020	Original guideline citation (given in bold numbers) and as listed in the eReference (superscripts)
**Claim 2**. Attaining no evidence of disease activity (NEDA), defined as no relapses, no EDSS worsening, and no MRI lesion activity, is an ideal goal but is difficult to achieve in practice. Most patients will not achieve NEDA even with a high-efficacy DMT **(86–90)** and the NEDA rate will invariably decline during the course of treatment **(91).** NEDA has been criticized as not reflecting the underlying pathophysiology of MS **(92),** and the predictive value of NEDA for long-term disability outcomes has been questioned **(93,94).** Thus, patients and clinicians will need to tolerate some minimal evidence of disease activity (MEDA), a term that has not been adequately defined. In studies to date, minimal evidence of MRI activity (< 3 new T2 or < 2 Gd+ lesions) was not predictive of EDSS worsening over a 6.7-year follow-up **(95).** However, a retrospective analysis reported that the long-term risk of disability (EDSS ≥ 6) was similar with NEDA and MEDA, when MEDA was defined as no relapses, <3 new T2 lesions, and no Gd+ lesions in the first year of treatment. **(96)** Additional studies using different definitions of MEDA are required.	**Claim in the Paper 86**[59]**:** This post-hoc study is not considered because it was conducted "to determine the effects of natalizumab compared with placebo on the proportion of patients who were free of disease activity over 2 years". No predictive statement is claimed for MRI activity (SEP) to predict CEP.<br><br>**Claim in the Paper 87**[60]**:** This post-hoc study is not considered because it was conducted "to evaluate the effect of fingolimod 0.5 mg on disease activity in young adults with MS from three randomized, double-blind Phase 3 trials". No predictive statement is claimed for MRI activity (SEP) to predict CEP.<br><br>**Claim in the Paper 88**[61]**:** As with sources 86-87, this paper is not considered, as no predictive claim was made for MRI activity (SEP) to predict CEP.<br><br>**Claim in the Paper 89**[62]**:** As with sources 86-87, this paper is not considered as no predictive claim was made for MRI activity (SEP) to predict CEP.<br><br>**Claim in the Paper 90**[63]**:** As with sources 86-87, this paper is not considered as no predictive claim was made for MRI activity (SEP) to predict CEP.
	**Claim in the Paper 91**[64]**:** PPVs and NPVs of NEDA and each component of NEDA for predicting the absence of progression (EDSS score change ≤0.5) at 7 years were calculated each year, and it was found that "NEDA at 2 years had a positive predictive value of 78.3% for no progression (Expanded Disability Status Scale score change ≤0.5) at 7 years".<br><br>**Our Assessment:** The study does not report PPVs for MRI alone and its CIs. Moreover, this paper states that the absence of disease activity, not the presence, can be predictive of no RRMS worsening: "NEDA status at 2 years may be optimal in terms of prognostic value in the longer term".<br><br>***Claim in the Paper 92***[65]***:*** "Whilst it is self-explanatory that occurrence of new MRI lesions principally reflects MS disease activity, a validated and reliable cut-off for prediction of long-term disability progression being the basis for treatment decision making has yet to be determined".<br><br>***Our Assessment***: This paper critically examines the concept of NEDA and offers an argument that largely aligns with our paper: the MRI lesion activity as the SEP still needs to be reliably assessed with relevant statistical tools.<br><br>***Claim in the Paper 93***[66]***:*** "Our findings suggest that the inclusion of MRI measures into the definition of NEDA lessens its predictive value". The results were as follows: "A total of 245 and 371 patients were evaluated at 16 and 21 years, respectively. Clinical NEDA predicted NDOs (Negative Disability Outcome) (p=0.0029), as did baseline EDSS (p < 0.0001), baseline T2-BOD (p < 0.0001), and change in T2-BOD (p = 0.0033). IFNB-1b treatment (p = 0.0251), relapse rate in the 2 years before study start (p = 0.0260), T2-BOD at

Claim made in CMSWG 2020	Original guideline citation (given in bold numbers) and as listed in the eReference (superscripts)
	baseline (p = 0.0014), and change in T2-BOD (p = 0.0129) predicted survival at 21 years".<br><br>**Our Assessment**: In this paper, "Associations were expressed both as hazard rates (HR) and odds ratios (OR), comparing patients who had achieved NEDA with those who hadn't". Thus, regression models with no PPVs or ROC were used. Moreover, this paper supports the point that MRI does not add to the predictive ability of NEDA.<br><br>**Claim in the Paper 94[67]:** "Subjects with no evidence of disease activity (NEDA) by clinical and MRI criteria during the first two years had long-term outcomes that were no different from those of the cohort as a whole", where "Logistic regression was used … to model clinical and MRI changes from baseline to year 2 and escalation therapy as predictor variables for long-term disability outcomes".<br><br>**Our Assessment:** This paper uses logistic regression and ORs to assess the predictive value of NEDA that includes MRI lesions. Moreover, the results from the paper show that NEDA, which includes MRI activity, is not a good predictor.
	**Paper 95[37]** has already been discussed in Supplementary Table 3 (Claim 2) as Source 85.<br><br>**Claim in the Paper 96[68]*:*** The study compared MEDA with traditional NEDA-3 and EDA (Evidence of Disease Activity). The authors found that disability worsening occurred in 17.3% of NEDA-3, 18.0% of MEDA, and 29.3% of EDA patients. The risk of CDW was significantly higher in EDA (HR = 1.74, 95% CI: 1.36–2.22), but not in MEDA (HR = 1.07, 95% CI: 0.78–1.46) compared to NEDA-3. Predictive values showed PPV for CDW was similar between NEDA-3 (17.3%, 95% CI: 14.5–20.1) and MEDA (18.0%, 95% CI: 13.4–22.5), and substantially higher in EDA (29.3%, 95% CI: 26.3–32.4).<br><br>**Our Assessment**: The study used hazard ratios and predictive values, offering evidence that mild MRI activity may not weaken the prognostic value of NEDA metrics. MRI alone, unless showing high activity, may not function as a strong surrogate for clinical outcomes. However, the PPVs are low even for EDA (29.3%).
**Claim 3.** Contrast enhancement with Gd is recommended for the initial and re-baseline scans. For the re-baseline scan, the presence of Gd-enhancing lesions may be useful to identify a suboptimal drug response earlier. Gd enhancement is helpful for monitoring clinically silent disease but is not routinely required for follow-up scans. Most enhancing lesions will have a T2 lesion	**Claim 1 made in the Source 113[69]:** "Determining ongoing radiologic stability is based on the presence or absence of new lesions (T2 or contrast-enhancing T1) relative to a posttreatment MR imaging (35)".<br><br>**Our Assessment**: This claim states that after the treatment initiation and comparing with post treatment MRI measurements if new lesions are present, radiological stability can be found.<br><br>**In Source 35[70]**, "The correlation coefficients (R2) with downstream disability for new/enlarging T2 lesions and brain atrophy were 0.61 and 0.48, respectively, with both measures retained in a final model with a combined R2 of 0.75, strongly supporting the use of these MR imaging outcomes as clinical surrogate measures when applied in an appropriate clinical-/treatment-specific context (9).<br><br>**In Source 9[71] of the Source 35[70]**, "A linear regression, weighted for trial size and duration, was used to assess the relationship between the

Claim made in CMSWG 2020	Original guideline citation (given in bold numbers) and as listed in the eReference (superscripts)
equivalent, T2 lesions are more predictive of outcomes, and the addition of Gd does not appear to provide much additional information **(114–116)**. Gd may be clinically useful if there is a concern about an alternative diagnosis or when confluent lesions preclude the detection of new lesions on T2-weighted imaging alone **(113)**.	treatment effects on MRI markers and on disability progression". Results: "Thirteen trials including >13,500 RRMS patients were included in the meta-analysis. Treatment effects on disability progression were correlated with treatment effects both on brain atrophy ($R^2 = 0.48$, $p = 0.001$) and on active MRI lesions ($R^2 = 0.61$, $p < 0.001$). When the effects on both MRI endpoints were included in a multivariate model, the correlation was higher ($R^2 = 0.75$, $p < 0.001$)". Although a correlation may hint at a useful relationship, it is insufficient to define a reliable surrogate.<br><br>**Claim 2 in the Source 113**[69]**:** "A follow-up MR imaging is useful for patients on a disease-modifying therapy to determine the response of subclinical disease activity to treatment. Continued or worsening of MR imaging disease activity while on a disease-modifying therapy may prompt a change in therapy. There is evolving evidence that ongoing MR imaging activity can be indicative of a suboptimal therapeutic response (36-41)".<br><br>**In Source 36**[72], "13-year longitudinal study in 30 patients" was conducted to "evaluate the relation between T2 lesions and disease severity" in RRMS. "To assess the association of each MRI parameter and its 2-year change with clinical and MRI outcomes 13 years later, we determined Pearson correlation coefficients". Although a correlation may hint at a useful relationship, it is insufficient to define a reliable surrogate.<br>**Source 37**[6] has been discussed in Source 19 in Supplementary Table 1 (Claim 1) as Source 106.<br>**Source 38**[21] has been discussed in Source 78 in Supplementary Table 1 (Claim 3) as Source 29<br>**Source 39**[45] will be discussed in Supplementary Table 7 as Source 98.<br>**Source 40**[22] has been discussed in Source 78 in Supplementary Table 1 (Claim 3) as Source 30.<br>**Source 41**[16] has been discussed as Source 67 in Supplementary Table 1 (Claim 3).<br><br>**Claim 3 in the Source 113**[69]**:** "New T2 or gadolinium-enhancing lesions are associated with progressive changes in normal appearing brain tissue and global brain atrophy **(42)**".<br><br>**Source 42**[73] has already been discussed as Source 124 in Supplementary Table 7.<br><br>**Claim 4 in the Source 113**[69]**:** "While guidelines on a tolerable threshold for new lesion activity that warrants a change in therapy have been proposed **(43,44)**, individual factors will impact the clinician's decision on the frequency of MR imaging monitoring.<br><br>**Source 43**[74] **has 2 claims:**<br>**Claim 1 in Source 43**: "The utility of frequent MRI monitoring of disease activity and response to therapy is still unclear. In a recently published six-year follow-up study, the factors associated with a positive response to ongoing immunotherapy include two MRI criteria as readout for efficacy, a low T1 lesion volume and lack of gadolinium uptake after one year of treatment [121]."<br><br>**In Source 121**[75], "The authors reviewed a series of 10 consecutive patients treated with plasma exchange (PE) for acute, severe optic neuritis (ON) largely unresponsive to previous high-dose IV glucocorticosteroids". Thus, this source does not study RRMS and MRI as a

Claim made in CMSWG 2020	Original guideline citation (given in bold numbers) and as listed in the eReference (superscripts)
	predictor or surrogate.<br><br>**Claim 2 in Source 43**[74]: If the recent medical history and the current clinical findings indicate a relevant change with new relapse activity, or if cognitive decline or other signs of clinical progression become evident, all indicating an insufficient treatment response [124], a repeat cranial and even spinal MRI should be done as an additional (surrogate) marker for disease activity and progression.<br><br>**Source 124**[23] has already been discussed in Source 78 in Supplementary Table 1 as Source 31.<br>**Source 44**[25] is the Canadian MS Working Group Recommendation 2013 which is reviewed separately in Table 3.
	**Claim in the Paper 114**[76]**:** "The main conclusion from the present study is that Gd-DTPA is a sensitive marker of the BBB impairment which occurs consistently in new plaques. As such it should prove useful in monitoring therapeutic trials in MS.".<br><br>**Our Assessment:** This study has a small sample size of 9 CDMS patients. The study measures whether "Gd-DTPA is a useful marker of new and biologically active lesions". No claims on prognostic and surrogate ability of MRI is made.
	**Claim in the Paper 115**[77]**:** "This study aimed to evaluate the relevance of GBCA [Gd-based Contrast Enhancement Agents] injections during the MRI follow-up of MS patients under natalizumab (Tysabri) treatment". "According to this study based on the clinical and radiological practice, the systematic use of GBCA seems of limited relevance in the MRI follow-up of asymptomatic patients treated continuously with natalizumab".<br><br>**Our Assessment:** The paper aims to assess GDBC. No claim on the predictive ability of MRI activity was made.
	**Claim in the Paper 116**[78]**:** The aim was "To investigate the proportion of cases in which MRI activity would be detectable only using contrast-enhanced T1-weighted sequences". The main conclusion of the paper is as follows: "Reactivation of pre-existing lesions is limited to a tiny fraction of MRI studies. Gd+T1-weighted images could be omitted, in patients treated with DMT for at least 6 months, without relevant loss of information".<br><br>**Our Assessment:** Although this study cites sources claiming that MRI lesion activity is a reliable predictor or surrogate for CEP, the study itself does not contain any claims on the predictive ability of MRI activity.
**Claim 4.** Recommendation 9. New/enlarging T2-weighted MRI lesions while on DMT are correlated with new relapses and clinical	**Source 95**[37] has already been discussed as Source 85 in Supplementary Table 3.
	**Claim in the Paper 98**[45]**:** This is a "multicenter, observational, 15-year follow-up study of patients who completed 2 years in the pivotal trial of IM IFNb-1a for RRMS", where "In the IM IFNb-1a group, persistent disease activity predicted severe EDSS worsening:

<table>
<tr><th>Claim made in CMSWG 2020</th><th>Original guideline citation (given in bold numbers) and as listed in the eReference (superscripts)</th></tr>
<tr><td rowspan="4">disability progression over time. A finding of ≥3 new/ enlarging lesions while on a DMT is considered a suboptimal response, and a change in treatment is recommended (Table 2). MRI is the most sensitive method for detecting ongoing CNS inflammation and provides a good indication of a suboptimal therapeutic response (95,98,99). Several studies have reported that ≥3 new/enlarging lesions in the first year of therapy are predictive of worsening EDSS scores during long-term follow-up (95,123). Accordingly, ≥3 new lesions during treatment would be a Major criterion (Table 2). However, any evidence of ongoing disease activity in a treated patient is worrisome, and the presence of fewer new/enlarging T2 lesions will still warrant early reassessment, particularly if accompanied by a relapse. T2 lesion load in the first 5 years has been shown to be more strongly correlated with brain atrophy (124).</td><td>gadolinium-enhancing lesions (odds ratio [OR], 8.96; p < 0.001); relapses (OR, 4.44; p=0.010); and new T2 lesions (OR, 2.90; p=0.080)”.

Our Assessment: In this study, “odds ratios were used to evaluate the ability of early disease activity markers to predict long-term EDSS progression into the worst quartile during the 15 years post-randomization”. Logistic regression and ORs are not sufficient to show a reliable predictor.</td></tr>
<tr><td>Claim in the Paper 99[79]: No claim on the predictive ability of MRI activity was made.</td></tr>
<tr><td>Claim in the Paper 123[80]: This study “analysed data from 392 patients starting IFNB and regularly followed up to 5 years”, where “The risk of relapses (year 2–5) was associated with … new T2-hyperintense lesions (for 2 new lesions: HR=1.96, p=0.011; for ≥3 new lesions: HR=3.55, p=0.001) in the first year of treatment … the risk of disability worsening (year 2–5) was associated with ≥2 relapses (HR=4.33, p=0.001) and new spinal cord or infratentorial lesions (HR=4.45,p=0.001) in the first year of treatment”.

Our Assessment: “Cox proportional hazards models (stepwise fashion) were built to investigate the best combination of demographic, clinical and MRI predictors”. which is not sufficient to find a reliable predictor. However, it is worth noting that this paper dives into not only the count and volume of the T2 lesions, but also the location. KM curves with no CIs are provided.</td></tr>
<tr><td>Claim in the Paper 124[73]: “28 subjects with clinically probable or definite multiple sclerosis were followed for 14 years after first onset of symptoms” to “explore the association between changing brain lesion loads and subsequent tissue atrophy”. Results: “Change in lesion load in the first five years was more closely correlated to disease related brain atrophy at 14 years than later changes in lesion load, although the correlation was only moderate (Spearman correlation = -0.528, p = 0.004)” and (Linear Regression = R^2 = 0.4363, p = 0.003)

Our Assessment: This study may have a low sample size and measures brain atrophy, not EDSS worsening, which is not a clinical endpoint. Moreover, this paper uses Spearman Rank Correlation and Linear Regression “to estimate the relative contribution of changing T2 lesion loads between the time points studied and estimated atrophy at the 14-year follow-up” to establish the predictive ability of brain lesions, which is not sufficient to find a reliable surrogate or a predictor.</td></tr>
</table>

Supplementary Figure 4. References Used in References of CMSWG 2020

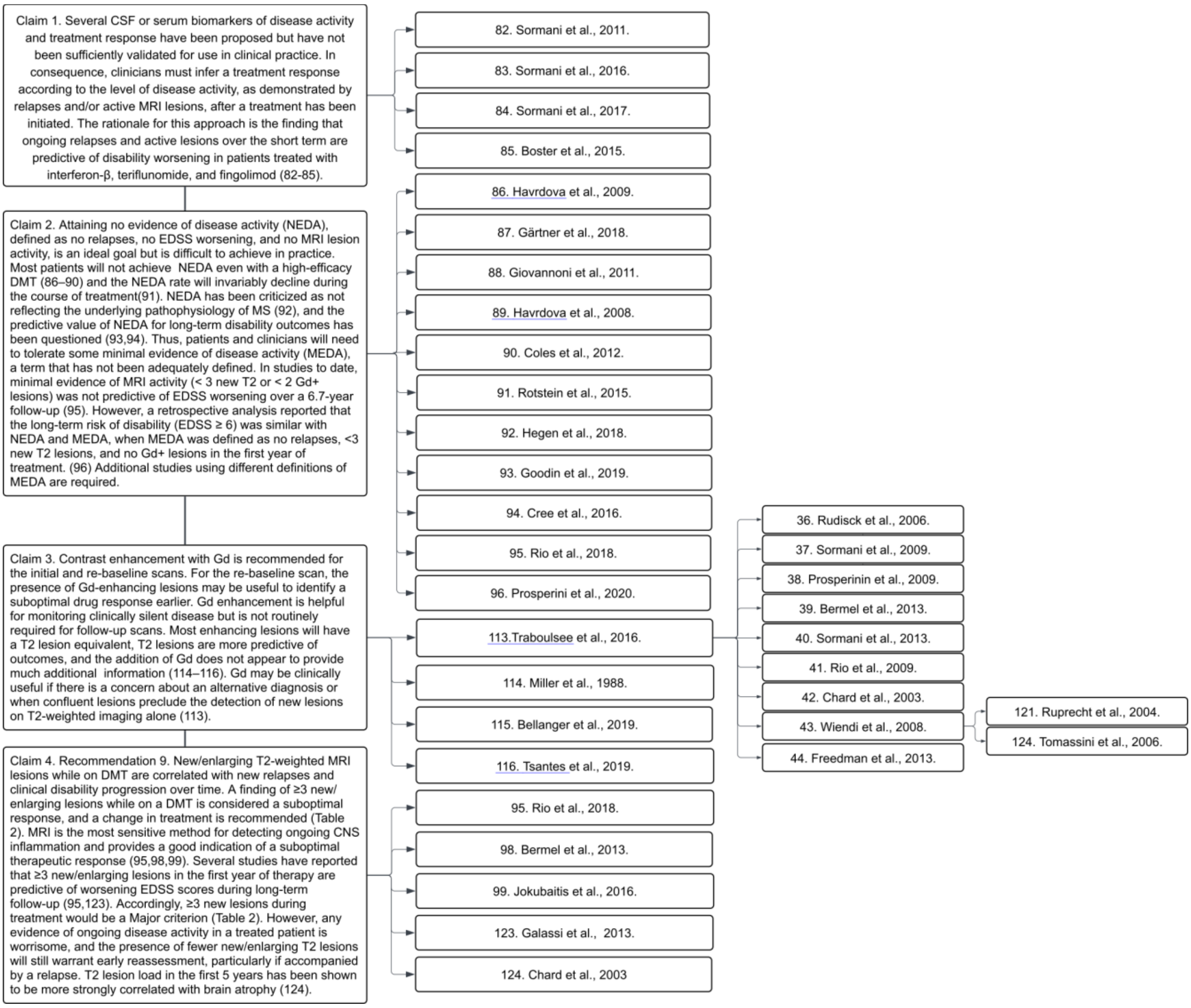

The diagram above shows the visual representation of citation flow in the CMSWG 2020 Guideline used as evidence for predictive ability of MRI markers. The first column represents statements from the guideline and the references used therein. The second column represent sources of evidence as cited in the guideline (first line literature), the third column refers to papers cited in first line literature.

Supplementary Table 8. Classifying the Evidence Strategies Used to Claim Predictive Value of MRI in CMSWG 2020[55]

Claim	Predictive Models	Group-specific predictive values (PPV, NPV, KM Curves as Indirect Information)	Sensitivity and Specificity	Prentice Criteria and PTE	OR, HR without model reported	Correlation	Comparing groups	Likelihood Ratio Test (LRT)	Not aimed to measure the predictive quality of MRI
Several CSF or serum biomarkers of disease activity and …		83[56] (KM Curves with no CIs and PPV reported), 84[57] (KM Curves with no CIs and PPV with no CIs reported)	83[56]	82[8]	83[56], 84[57], 85[58]		83[56], 84[57]		85[58](confe-rence abstract) 86[59]
Attaining no evidence of disease activity (NEDA), defined …		91[64] (PPV reported with no CIs), 96[68] (KM Curves with no CIs and PPV reported)	96[68]		93[66], 94[67], 95[38], 96[68]		91[64], 96[68]		86[59], 87[60], 88[61], 89[62], 90[63], 91[65] (Review),
Contrast enhancement with Gd is recommended for the initial…		113[69](38[21]) (PPV reported) 113[69][40[22]] (KM curves reported)	113[69][38[21]]		113[69][39[45]], 113[69][40[22]], 113[69][41[16]], 113[69][38[21]], 43[74][44[23]]	113[69][35[70][9[71]]], 113[69][36[72]], 113[69][37[6]], 113[69][42[73]]	43[74][44[23]], 113[69][38[21]], 113[69][36[72]]	113[69][38[21]]	43[74][121[75]], 114[76], 115[77], 116[78]
Recommendation 9. New/enlarging T2-weighted MRI lesions…		95[37] (PPV with no CIs reported), 123[80] (KM Curves with no CIs reported)	95[37]		95[37], 98[45], 123[80]	124[73]	123[80]		99[79]

The table classifies the references discussed in Supplementary Table 7 according to methodological aspects. Numbers refer to the references in the guideline, superscripts to the Supplementary References. Numbers in square brackets represent the sources as cited in the first-line literature.

Supplementary References

1. Mayring P. Qualitative Content Analysis: Theoretical Background and Procedures. In: Bikner-Ahsbahs A, Knipping C, Presmeg N, eds. *Approaches to Qualitative Research in Mathematics Education*. Advances in Mathematics Education. Springer Netherlands; 2015:365-380. doi:10.1007/978-94-017-9181-6_13

2. Wattjes MP, Rovira À, Miller D, et al. Evidence-based guidelines: MAGNIMS consensus guidelines on the use of MRI in multiple sclerosis--establishing disease prognosis and monitoring patients. *Nat Rev Neurol*. 2015;11(10):597-606. doi:10.1038/nrneurol.2015.157

3. Simon JH, Li D, Traboulsee A, et al. Standardized MR imaging protocol for multiple sclerosis: Consortium of MS Centers consensus guidelines. *AJNR Am J Neuroradiol*. 2006;27(2):455-461.

4. Brex PA, Ciccarelli O, O'Riordan JI, Sailer M, Thompson AJ, Miller DH. A longitudinal study of abnormalities on MRI and disability from multiple sclerosis. *N Engl J Med*. 2002;346(3):158-164. doi:10.1056/NEJMoa011341

5. Cook SD, Dhib-Jalbut S, Dowling P, et al. Use of Magnetic Resonance Imaging as Well as Clinical Disease Activity in the Clinical Classification of Multiple Sclerosis and Assessment of Its Course: A Report from an International CMSC Consensus Conference, March 5-7, 2010. *Int J MS Care*. 2012;14(3):105-114. doi:10.7224/1537-2073-14.3.105

6. Sormani MP, Bonzano L, Roccatagliata L, Cutter GR, Mancardi GL, Bruzzi P. Magnetic resonance imaging as a potential surrogate for relapses in multiple sclerosis: a meta-analytic approach. *Ann Neurol*. 2009;65(3):268-275. doi:10.1002/ana.21606

7. Sormani MP, Bonzano L, Roccatagliata L, Mancardi GL, Uccelli A, Bruzzi P. Surrogate endpoints for EDSS worsening in multiple sclerosis. A meta-analytic approach. *Neurology*. 2010;75(4):302-309. doi:10.1212/WNL.0b013e3181ea15aa

8. Sormani MP, Li DK, Bruzzi P, et al. Combined MRI lesions and relapses as a surrogate for disability in multiple sclerosis. *Neurology*. 2011;77(18):1684-1690. doi:10.1212/WNL.0b013e31823648b9

9. Sormani MP, Stubinski B, Cornelisse P, Rocak S, Li D, De Stefano N. Magnetic resonance active lesions as individual-level surrogate for relapses in multiple sclerosis. *Mult Scler Houndmills Basingstoke Engl*. 2011;17(5):541-549. doi:10.1177/1352458510391837

10. Prentice RL. Surrogate endpoints in clinical trials: definition and operational criteria. *Stat Med*. 1989;8(4):431-440. doi:10.1002/sim.4780080407

11. Verhey LH, Narayanan S, Banwell B. Standardized magnetic resonance imaging acquisition and reporting in pediatric multiple sclerosis. *Neuroimaging Clin N Am*. 2013;23(2):217-226.e1-7. doi:10.1016/j.nic.2012.12.003

12. Barkhof F, Simon JH, Fazekas F, et al. MRI monitoring of immunomodulation in relapse-onset multiple sclerosis trials. *Nat Rev Neurol*. 2011;8(1):13-21. doi:10.1038/nrneurol.2011.190

13. Sormani MP, Bruzzi P, Comi G, Filippi M. MRI metrics as surrogate markers for clinical relapse rate in relapsing-remitting MS patients. *Neurology*. 2002;58(3):417-421. doi:10.1212/wnl.58.3.417

14. Sormani MP, Bruzzi P, Beckmann K, et al. MRI metrics as surrogate endpoints for EDSS progression in SPMS patients treated with IFN beta-1b. *Neurology*. 2003;60(9):1462-1466. doi:10.1212/01.wnl.0000063312.15758.b3

15. Petkau J, Reingold S, Held U, et al. Magnetic resonance imaging as a surrogate outcome for multiple sclerosis relapses. *Mult Scler Houndmills Basingstoke Engl*. 2008;14(6):770-778. doi:10.1177/1352458507088104

16. Río J, Castilló J, Rovira A, et al. Measures in the first year of therapy predict the response to interferon beta in MS. *Mult Scler Houndmills Basingstoke Engl*. 2009;15(7):848-853. doi:10.1177/1352458509104591

17. Sormani MP, De Stefano N. Defining and scoring response to IFN-β in multiple sclerosis. *Nat Rev Neurol*. 2013;9(9):504-512. doi:10.1038/nrneurol.2013.146

18. Wang YC, Sandrock A, Richert JR, Meyerson L, Miao X. Short-Term Relapse Quantitation as a Fully Surrogate Endpoint for Long-Term Sustained Progression of Disability in RRMS Patients Treated with Natalizumab. *Neurol Res Int*. 2011;2011:195831. doi:10.1155/2011/195831

19. Durelli L, Barbero P, Bergui M, et al. MRI activity and neutralising antibody as predictors of response to interferon β treatment in multiple sclerosis. *J Neurol Neurosurg Psychiatry*. 2008;79(6):646-651. doi:10.1136/jnnp.2007.130229

20. Río J, Rovira A, Tintoré M, et al. Relationship between MRI lesion activity and response to IFN-beta in relapsing-remitting multiple sclerosis patients. *Mult Scler Houndmills Basingstoke Engl*. 2008;14(4):479-484. doi:10.1177/1352458507085555

21. Prosperini L, Gallo V, Petsas N, Borriello G, Pozzilli C. One-year MRI scan predicts clinical response to interferon beta in multiple sclerosis. *Eur J Neurol*. 2009;16(11):1202-1209. doi:10.1111/j.1468-1331.2009.02708.x

22. Sormani MP, Rio J, Tintorè M, et al. Scoring treatment response in patients with relapsing multiple sclerosis. *Mult Scler Houndmills Basingstoke Engl*. 2013;19(5):605-612. doi:10.1177/1352458512460605

23. Tomassini V, Paolillo A, Russo P, et al. Predictors of long-term clinical response to interferon beta therapy in relapsing multiple sclerosis. *J Neurol*. 2006;253(3):287-293. doi:10.1007/s00415-005-0979-5

24. Prosperini L, Mancinelli CR, De Giglio L, De Angelis F, Barletta V, Pozzilli C. Interferon beta failure predicted by EMA criteria or isolated MRI activity in multiple sclerosis. *Mult Scler J*. 2014;20(5):566-576. doi:10.1177/1352458513502399

25. Freedman MS, Selchen D, Arnold DL, et al. Treatment Optimization in MS: Canadian MS Working Group Updated Recommendations. *Can J Neurol Sci*. 2013;40(3):307-323. doi:10.1017/S0317167100014244

26. Stangel M, Penner IK, Kallmann BA, Lukas C, Kieseier BC. Towards the implementation of 'no evidence of disease activity' in multiple sclerosis treatment: the multiple sclerosis decision model. *Ther Adv Neurol Disord*. 2015;8(1):3-13. doi:10.1177/1756285614560733

27. Barkhof F, Scheltens P, Frequin ST, et al. Relapsing-remitting multiple sclerosis: sequential enhanced MR imaging vs clinical findings in determining disease activity. *AJR Am J Roentgenol*. 1992;159(5):1041-1047. doi:10.2214/ajr.159.5.1414773

28. Weiner HL, Guttmann CR, Khoury SJ, et al. Serial magnetic resonance imaging in multiple sclerosis: correlation with attacks, disability, and disease stage. *J Neuroimmunol*. 2000;104(2):164-173. doi:10.1016/s0165-5728(99)00273-8

29. Rudick RA, Lee JC, Simon J, Ransohoff RM, Fisher E. Defining interferon beta response status in multiple sclerosis patients. *Ann Neurol*. 2004;56(4):548-555. doi:10.1002/ana.20224

30. Visser F, Wattjes MP, Pouwels PJW, Linssen WHJP, van Oosten BW. Tumefactive multiple sclerosis lesions under fingolimod treatment. *Neurology*. 2012;79(19):2000-2003. doi:10.1212/WNL.0b013e3182735cb3

31. Pilz G, Harrer A, Wipfler P, et al. Tumefactive MS lesions under fingolimod: a case report and literature review. *Neurology*. 2013;81(19):1654-1658. doi:10.1212/01.wnl.0000435293.34351.11

32. Wattjes MP, Ciccarelli O, Reich DS, et al. 2021 MAGNIMS–CMSC–NAIMS consensus recommendations on the use of MRI in patients with multiple sclerosis. *Lancet Neurol*. 2021;20(8):653-670. doi:10.1016/S1474-4422(21)00095-8

33. Rae-Grant A, Day GS, Marrie RA, et al. Practice guideline recommendations summary: Disease-modifying therapies for adults with multiple sclerosis: Report of the Guideline Development, Dissemination, and Implementation Subcommittee of the American Academy of Neurology. *Neurology*. 2018;90(17):777-788. doi:10.1212/wnl.0000000000005347

34. Fisniku LK, Brex PA, Altmann DR, et al. Disability and T2 MRI lesions: a 20-year follow-up of patients with relapse onset of multiple sclerosis. *Brain J Neurol*. 2008;131(Pt 3):808-817. doi:10.1093/brain/awm329

35. Gasperini C, Prosperini L, Tintoré M, et al. 67 Unraveling treatment response in multiple sclerosis: A clinical and MRI challenge. *Neurology*. 2019;92(4):180-192. doi:10.1212/WNL.0000000000006810

36. Signori A, Schiavetti I, Gallo F, Sormani MP. Subgroups of multiple sclerosis patients with larger treatment benefits: a meta-analysis of randomized trials. *Eur J Neurol*. 2015;22(6):960-966. doi:10.1111/ene.12690

37. Río J, Rovira À, Tintoré M, et al. Disability progression markers over 6-12 years in interferon-β-treated multiple sclerosis patients. *Mult Scler Houndmills Basingstoke Engl*. 2018;24(3):322-330. doi:10.1177/1352458517698052

38. Bovis F, Carmisciano L, Signori A, et al. Defining responders to therapies by a statistical modeling approach applied to randomized clinical trial data. *BMC Med*. 2019;17(1):113. doi:10.1186/s12916-019-1345-2

39. Zhao L, Tian L, Cai T, Claggett B, Wei LJ. EFFECTIVELY SELECTING A TARGET POPULATION FOR A FUTURE COMPARATIVE STUDY. *J Am Stat Assoc*. 2013;108(502):527-539. doi:10.1080/01621459.2013.770705

40. Marta M, Giovannoni G. Disease modifying drugs in multiple sclerosis: mechanisms of action and new drugs in the horizon. *CNS Neurol Disord Drug Targets*. 2012;11(5):610-623. doi:10.2174/187152712801661301

41. Comi G, Filippi M, Wolinsky JS. European/Canadian multicenter, double-blind, randomized, placebo-controlled study of the effects of glatiramer acetate on magnetic resonance imaging--measured disease activity and burden in patients with relapsing multiple sclerosis. European/Canadian Glatiramer Acetate Study Group. *Ann Neurol*. 2001;49(3):290-297.

42. Coles AJ, Fox E, Vladic A, et al. Alemtuzumab more effective than interferon β-1a at 5-year follow-up of CAMMS223 clinical trial. *Neurology*. 2012;78(14):1069-1078. doi:10.1212/WNL.0b013e31824e8ee7

43. Río J, Auger C, Rovira À. MR Imaging in Monitoring and Predicting Treatment Response in Multiple Sclerosis. *Neuroimaging Clin N Am*. 2017;27(2):277-287. doi:10.1016/j.nic.2017.01.001

44. Tintore M, Rovira À, Río J, et al. Defining high, medium and low impact prognostic factors for developing multiple sclerosis. *Brain J Neurol*. 2015;138(Pt 7):1863-1874. doi:10.1093/brain/awv105

45. Bermel RA, You X, Foulds P, et al. Predictors of long-term outcome in multiple sclerosis patients treated with interferon β. *Ann Neurol*. 2013;73(1):95-103. doi:10.1002/ana.23758

46. Río J, Comabella M, Montalban X. Predicting responders to therapies for multiple sclerosis. *Nat Rev Neurol*. 2009;5(10):553-560. doi:10.1038/nrneurol.2009.139

47. Paty DW, Li DK. Interferon beta-1b is effective in relapsing-remitting multiple sclerosis. II. MRI analysis results of a multicenter, randomized, double-blind, placebo-controlled trial. UBC MS/MRI Study Group and the IFNB Multiple Sclerosis Study Group. *Neurology*. 1993;43(4):662-667. doi:10.1212/wnl.43.4.662

48. Simon JH, Jacobs LD, Campion M, et al. Magnetic resonance studies of intramuscular interferon beta-1a for relapsing multiple sclerosis. The Multiple Sclerosis Collaborative Research Group. *Ann Neurol*. 1998;43(1):79-87. doi:10.1002/ana.410430114

49. Li DK, Paty DW. Magnetic resonance imaging results of the PRISMS trial: a randomized, double-blind, placebo-controlled study of interferon-beta1a in relapsing-remitting multiple sclerosis. Prevention of Relapses and Disability by Interferon-beta1a Subcutaneously in Multiple Sclerosis. *Ann Neurol*. 1999;46(2):197-206. doi:10.1002/1531-8249(199908)46:2%3C197::aid-ana9%3E3.0.co;2-p

50. Sormani MP, Rovaris M, Comi G, Filippi M. A composite score to predict short-term disease activity in patients with relapsing-remitting MS. *Neurology*. 2007;69(12):1230-1235. doi:10.1212/01.wnl.0000276940.90309.15

51. Pozzilli C, Prosperini L, Sbardella E, De Giglio L, Onesti E, Tomassini V. Post-marketing survey on clinical response to interferon beta in relapsing multiple sclerosis: the Roman experience. *Neurol Sci Off J Ital Neurol Soc Ital Soc Clin Neurophysiol*. 2005;26 Suppl 4:S174-178. doi:10.1007/s10072-005-0510-x

52. Riddell CA, Zhao Y, Li DKB, et al. Evaluation of safety monitoring guidelines based on MRI lesion activity in multiple sclerosis. *Neurology*. 2011;77(24):2089-2096. doi:10.1212/WNL.0b013e31823d762d

53. Randomised double-blind placebo-controlled study of interferon beta-1a in relapsing/remitting multiple sclerosis. PRISMS (Prevention of Relapses and Disability by Interferon beta-1a Subcutaneously in Multiple Sclerosis) Study Group. *Lancet Lond Engl*. 1998;352(9139):1498-1504.

54. Polman CH, Reingold SC, Banwell B, et al. Diagnostic criteria for multiple sclerosis: 2010 revisions to the McDonald criteria. *Ann Neurol*. 2011;69(2):292-302. doi:10.1002/ana.22366

55. Freedman MS, Devonshire V, Duquette P, et al. Treatment Optimization in Multiple Sclerosis: Canadian MS Working Group Recommendations. *Can J Neurol Sci*. 2020;47(4):437-455. doi:10.1017/cjn.2020.66

56. Sormani MP, Gasperini C, Romeo M, et al. Assessing response to interferon-β in a multicenter dataset of patients with MS. *Neurology*. 2016;87(2):134-140. doi:10.1212/WNL.0000000000002830

57. Sormani MP, Truffinet P, Thangavelu K, Rufi P, Simonson C, De Stefano N. Predicting long-term disability outcomes in patients with MS treated with teriflunomide in TEMSO. *Neurol Neuroimmunol Neuroinflammation*. 2017;4(5):e379. doi:10.1212/NXI.0000000000000379

58. Boster A, Hawker K, Ritter S, Tomic D, Sprenger T. Disease activity in the first year predicts longer-term clinical outcomes in the pooled population of the phase III FREEDOMS and FREEDOMS II studies (P7.239). *Neurology*. 2015;84(14_supplement):P7.239. doi:10.1212/WNL.84.14_supplement.P7.239

59. Havrdova E, Galetta S, Hutchinson M, et al. Effect of natalizumab on clinical and radiological disease activity in multiple sclerosis: a retrospective analysis of the Natalizumab Safety and Efficacy in Relapsing-Remitting Multiple Sclerosis (AFFIRM) study. *Lancet Neurol*. 2009;8(3):254-260. doi:10.1016/S1474-4422(09)70021-3

60. Gärtner J, Chitnis T, Ghezzi A, et al. Relapse Rate and MRI Activity in Young Adult Patients With Multiple Sclerosis: A Post Hoc Analysis of Phase 3 Fingolimod Trials. *Mult Scler J - Exp Transl Clin*. 2018;4(2):2055217318778610. doi:10.1177/2055217318778610

61. Giovannoni G, Cook S, Rammohan K, et al. Sustained disease-activity-free status in patients with relapsing-remitting multiple sclerosis treated with cladribine tablets in the CLARITY study: a post-hoc and subgroup analysis. *Lancet Neurol*. 2011;10(4):329-337. doi:10.1016/S1474-4422(11)70023-0

62. Havrdová E, Arnold DL, Bar-Or A, et al. No evidence of disease activity (NEDA) analysis by epochs in patients with relapsing multiple sclerosis treated with ocrelizumab vs interferon beta-1a. *Mult Scler J - Exp Transl Clin*. 2018;4(1):2055217318760642. doi:10.1177/2055217318760642

63. Coles AJ, Twyman CL, Arnold DL, et al. Alemtuzumab for patients with relapsing multiple sclerosis after disease-modifying therapy: a randomised controlled phase 3 trial. *Lancet Lond Engl*. 2012;380(9856):1829-1839. doi:10.1016/S0140-6736(12)61768-1

64. Rotstein DL, Healy BC, Malik MT, Chitnis T, Weiner HL. Evaluation of No Evidence of Disease Activity in a 7-Year Longitudinal Multiple Sclerosis Cohort. *JAMA Neurol*. 2015;72(2):152-158. doi:10.1001/jamaneurol.2014.3537

65. Hegen H, Bsteh G, Berger T. "No evidence of disease activity" - is it an appropriate surrogate in multiple sclerosis? *Eur J Neurol*. 2018;25(9):1107-e101. doi:10.1111/ene.13669

66. Goodin DS, Reder AT, Traboulsee AL, et al. Predictive validity of NEDA in the 16- and 21-year follow-up from the pivotal trial of interferon beta-1b. *Mult Scler J*. 2019;25(6):837-847. doi:10.1177/1352458518773511

67. University of California, San Francisco MS-EPIC Team:, Cree BAC, Gourraud PA, et al. Long-term evolution of multiple sclerosis disability in the treatment era. *Ann Neurol*. 2016;80(4):499-510. doi:10.1002/ana.24747

68. Prosperini L, Mancinelli C, Haggiag S, et al. Minimal evidence of disease activity (MEDA) in relapsing-remitting multiple sclerosis. *J Neurol Neurosurg Psychiatry*. 2020;91(3):271-277. doi:10.1136/jnnp-2019-322348

69. Traboulsee A, Simon JH, Stone L, et al. Revised Recommendations of the Consortium of MS Centers Task Force for a Standardized MRI Protocol and Clinical Guidelines for the Diagnosis and Follow-Up of Multiple Sclerosis. *AJNR Am J Neuroradiol*. 2016;37(3):394-401. doi:10.3174/ajnr.A4539

70. Simon JH, Bermel RA, Rudick RA. Simple MRI metrics contribute to optimal care of the patient with multiple sclerosis. *AJNR Am J Neuroradiol*. 2014;35(5):831-832. doi:10.3174/ajnr.A3937

71. Sormani MP, Arnold DL, De Stefano N. Treatment effect on brain atrophy correlates with treatment effect on disability in multiple sclerosis. *Ann Neurol*. 2014;75(1):43-49. doi:10.1002/ana.24018

72. Rudick RA, Lee JC, Simon J, Fisher E. Significance of T2 lesions in multiple sclerosis: A 13-year longitudinal study. *Ann Neurol*. 2006;60(2):236-242. doi:10.1002/ana.20883

73. Chard DT, Brex PA, Ciccarelli O, et al. The longitudinal relation between brain lesion load and atrophy in multiple sclerosis: a 14 year follow up study. *J Neurol Neurosurg Psychiatry*. 2003;74(11):1551-1554. doi:10.1136/jnnp.74.11.1551

74. Multiple Sclerosis Therapy Consensus Group (MSTCG), Wiendl H, Toyka KV, et al. Basic and escalating immunomodulatory treatments in multiple sclerosis: current therapeutic recommendations. *J Neurol*. 2008;255(10):1449-1463. doi:10.1007/s00415-008-0061-1

75. Ruprecht K, Klinker E, Dintelmann T, Rieckmann P, Gold R. Plasma exchange for severe optic neuritis: treatment of 10 patients. *Neurology*. 2004;63(6):1081-1083. doi:10.1212/01.wnl.0000138437.99046.6b

76. Miller DH, Rudge P, Johnson G, et al. Serial gadolinium enhanced magnetic resonance imaging in multiple sclerosis. *Brain J Neurol*. 1988;111 (Pt 4):927-939. doi:10.1093/brain/111.4.927

77. Bellanger G, Biotti D, Patsoura S, et al. What is the Relevance of the Systematic Use of Gadolinium During the MRI Follow-Up of Multiple Sclerosis Patients Under Natalizumab? *Clin Neuroradiol*. 2020;30(3):553-558. doi:10.1007/s00062-019-00794-0

78. Tsantes E, Curti E, Ganazzoli C, et al. The contribution of enhancing lesions in monitoring multiple sclerosis treatment: is gadolinium always necessary? *J Neurol*. 2020;267(9):2642-2647. doi:10.1007/s00415-020-09894-1

79. Jokubaitis VG, Spelman T, Kalincik T, et al. Predictors of long-term disability accrual in relapse-onset multiple sclerosis. *Ann Neurol*. 2016;80(1):89-100. doi:10.1002/ana.24682

80. Galassi S, Prosperini L, Logoteta A, et al. A lesion topography-based approach to predict the outcomes of patients with multiple sclerosis treated with Interferon Beta. *Mult Scler Relat Disord*. 2016;8:99-106. doi:10.1016/j.msard.2016.05.012
